# A Study of the Vaginal Microbiome in Healthy Canadian Women Utilizing *cpn*60-Based Molecular Profiling Reveals Distinct *Gardnerella* Subgroup Community State Types

**DOI:** 10.1371/journal.pone.0135620

**Published:** 2015-08-12

**Authors:** Arianne Y. K. Albert, Bonnie Chaban, Emily C. Wagner, John J. Schellenberg, Matthew G. Links, Julie van Schalkwyk, Gregor Reid, Sean M. Hemmingsen, Janet E. Hill, Deborah Money, VOGUE Research Group

**Affiliations:** 1 Women’s Health Research Institute, Vancouver, BC, Canada; 2 Department of Veterinary Microbiology, University of Saskatchewan, Saskatoon, SK, Canada; 3 Agriculture and AgriFood Canada, Saskatoon, SK, Canada; 4 Department of Obstetrics and Gynaecology, University of British Columbia, Vancouver, BC, Canada; 5 Department of Microbiology and Immunology, University of Western Ontario and Lawson Health Research Institute, London, ON, Canada; 6 National Research Council Canada, Saskatoon, SK, Canada; 7 Department of Microbiology & Immunology, University of Saskatchewan, Saskatoon, SK, Canada; 8 Faculty of Natural Sciences, Department of Life Sciences, Imperial College London, South Kensington Campus, London, United Kingdom; Fred Hutchinson Cancer Center, UNITED STATES

## Abstract

The vaginal microbiota is important in women’s reproductive and overall health. However, the relationships between the structure, function and dynamics of this complex microbial community and health outcomes remain elusive. The objective of this study was to determine the phylogenetic range and abundance of prokaryotes in the vaginal microbiota of healthy, non-pregnant, ethnically diverse, reproductive-aged Canadian women. Socio-demographic, behavioural and clinical data were collected and vaginal swabs were analyzed from 310 women. Detailed profiles of their vaginal microbiomes were generated by pyrosequencing of the chaperonin-60 universal target. Six community state types (CST) were delineated by hierarchical clustering, including three *Lactobacillus*-dominated CST (*L*. *crispatus*, *L*. *iners*, *L*. *jensenii*), two *Gardnerella-*dominated (subgroups A and C) and an “intermediate” CST which included a small number of women with microbiomes dominated by seven other species or with no dominant species but minority populations of *Streptococcus*, *Staphylococcus*, *Peptoniphilus*, *E*. *coli* and various Proteobacteria in co-dominant communities. The striking correspondence between Nugent score and deep sequencing CST continues to reinforce the basic premise provided by the simpler Gram stain method, while additional analyses reveal detailed *cpn*60-based phylogeny and estimated abundance in microbial communities from vaginal samples. Ethnicity was the only demographic or clinical characteristic predicting CST, with differences in Asian and White women (p = 0.05). In conclusion, this study confirms previous work describing four *cpn*60-based subgroups of *Gardnerella*, revealing previously undescribed CST. The data describe the range of bacterial communities seen in Canadian women presenting with no specific vaginal health concerns, and provides an important baseline for future investigations of clinically important cohorts.

## Introduction

The microorganisms that inhabit the human body, the microbiota, are an integral component of an individual’s health. Within the female reproductive tract, the resident vaginal microbiota play an important protectiv e role by interfering with the proliferation of organisms that cause vulvo-vaginal, urinary tract and sexually transmitted infections [1–5]. A healthy vaginal microbial community is generally defined as being dominated by *Lactobacillus* species, many of which produce acid, hydrogen peroxide, biosurfactants and bacteriocins antagonistic to pathogens [6–11]. Although the relationship between vaginal microbiology and adverse clinical symptoms (such as odour and discharge) is not well understood, women with a high diversity of anaerobic bacteria in communities dominated by organisms other than *Lactobacillus*, are generally considered to have a condition called bacterial vaginosis (BV). However, research especially on microbiota of asymptomatic women who meet the microbiological criteria of BV has expanded our understanding of the microbial constituents of the vagina [12–14].

High-throughput, culture-independent technologies have allowed investigation of the vaginal microbiome on a much larger scale and with unprecedented taxonomic resolution. To date, several research groups have surveyed the vaginal microbiomes of women from around the world in both cross-sectional [15–26] and longitudinal studies [27–33]. While methodologies and study cohorts vary considerably, the common finding from these studies has been that vaginal microbiota generally exist in one of a limited number of community configurations. These configurations have been termed “community state types” (CST) [17, 18, 27], with the most widely accepted typing scheme proposed by Ravel et al. [18], describing six CST depending on the dominant organisms present. CST I, II, III and V are *Lactobacillus*-dominated communities, composed primarily of *L*. *crispatus*, *L*. *gasseri*, *L*. *iners* or *L*. *jensenii*, respectively. CST IV was described initially as a heterogeneous group of non-lactobacilli [18] and has since been divided into two subgroups: IVA, consisting primarily of *Bifidobacterium*, *Dialister*, *Streptococcus*, and *Bacteroides*, and IVB containing more *Gardnerella*, *Prevotella*, *Megasphaera*, bacterial vaginosis-associated bacteria (BVAB), and *Mobiluncus* [27]. Other less frequently described vaginal CST include those where two *Lactobacillus* species co-dominate (such as *L*. *crispatus/L*. *iners* or *L*. *crispatus/L*. *jensenii*) or study-specific versions of the heterogeneous, non-lactobacilli CST IV [15–17, 19].

In addition to a growing consensus regarding the most commonly observed CST, an expanding list of individual factors and behaviours have been associated with particular CST or with women changing from a *Lactobacillus*-dominated CST to one associated with BV or *vice versa*. Profiles of vaginal microbiota have been reported to be associated with race/ethnicity [15, 18], level of education [34], use of hormonal contraceptives [35, 36], use of feminine hygiene products [37], gender of sexual partners [35, 38], number of sexual partners [39], condom use [35, 39], sexual behaviours [39] and smoking [38]. Some factors, such as level of education, most likely represent a composite variable of numerous factors associated with differences in the vaginal microbiota, including race/ethnicity, sexual behaviour and socioeconomic class [34]. Given the complex and interconnected nature of many of these factors, more research is needed with diverse cohorts of women to help clarify and understand how they contribute to the composition and dynamics of the vaginal microbiota.

In this study, phylogenetic profiles of vaginal microbiota were generated by massively parallel sequencing of the universal target (UT) from the *cpn*60 gene. This target has been shown to provide comparable information to the 16S rRNA gene in terms of community coverage [21] but provide better discrimination of species and subspecies [40–43], increased coverage of Bifidobacteriales [44, 45] and detection of eukaryotic microbes [46]. This resolution is critical, particularly for the vaginal microbiome, given recent evidence that the species *Gardnerella vaginalis* is actually comprised of four genotypically and phenotypically distinct subgroups, easily distinguished by variation in *cpn*60 UT sequences [47–49]. However, it is a recognized limitation of *cpn*60 that some Mollicutes, notably some species of *Mycoplasma* and *Ureaplasma*, lack this gene target. Given the established importance of these species in the vaginal microbiome, specific PCR assays were included in this study to estimate prevalence of these organisms. Finally, an estimate of total bacterial population density was generated using a 16S rRNA targeted quantitative PCR. The combination of *cpn*60 microbial profiling with targeted additional assays ensured complete microbiome profiling consistent with the largest 16S rRNA gene studies, but at a finer resolution with consequently increased phylogenetic detail.

The primary objective for this study was to define the range of vaginal microbiota profiles common in non-pregnant Canadian women of reproductive age without specific vaginal health concerns, in relation to socio-demographic, behavioural and clinical characteristics. In addition to our results broadly confirming previous studies in terms of the most commonly observed *Lactobacillus*-dominated CST in healthy women, they provide important phylogenetic insight into non-*Lactobacillus*-dominated CST based on distinct *cpn*60-based *Gardnerella* subgroups.

## Materials and Methods

### Ethics statement

This study received ethical approval from the University of British Columbia Children’s & Women’s Research Ethics Board (certificate no. H10-02535).

### Participants and study design

Healthy, reproductive-aged women aged 18–49 years were recruited from the greater Vancouver area of British Columbia, Canada, through research clinics, primary care offices, and online and print advertisements. Women were eligible to participate if the following inclusion criteria were met: between the ages of 18–49 and premenopausal (e.g. reported having a menstrual cycle over the previous 12 months), not pregnant, HIV negative, and had not used any oral or intravaginal antibiotic or antifungal treatments in the four weeks prior to the study visit (antiviral medications, e.g. valacyclovir for suppression of HSV infection, were permitted). After obtaining written informed consent, research staff collected demographic and clinical data from the participants via interview and by reviewing medical charts. Two vaginal swab samples were collected for Gram stain assessment and sequencing analysis, either by a clinician during an indicated speculum examination (i.e. routine pap smear screening), or by a study nurse conducting a speculum examination for study purposes, or using a validated self-collection method [28]. Amies gel transport swabs without charcoal (Stevens Company Ltd., Brampton, ON) were used to collect samples for Gram stain assessment, and dry Dacron swabs (Copan Diagnostics Inc., Murrieta, CA) were used to collect samples for sequencing analyses.

Samples collected for Gram stain assessment were processed within 48 h at the clinical laboratory for BC Women’s Hospital and Health Centre utilizing validated Nugent’s scoring methodology [50].

Samples collected for sequencing analyses were transferred to -80°C storage within 30 minutes, with the exception of those collected in community-based clinics, in which case, they were stored at 4°C for a maximum of 12 hours prior to transfer to -80°C. Total nucleic acid was extracted from batches of swabs using the MagMAX Total Nucleic Acid Isolation Kit (Applied Biosystems, Life Technologies, Burlington, ON, Canada) as per manufacturer’s instructions. To minimize opportunities for cross contamination between samples, small batches were processed. In addition, a negative control sample (sterile water) was run with each kit and tested with the subsequent *cpn*60 PCR to ensure no PCR product was generated.

### Quantitative PCR (qPCR) and conventional PCR

Samples were quantified for total bacterial DNA with a SYBR Green assay targeting the 16S rRNA gene (V3 region) as described previously [51]. Mollicutes (*Mycoplasma* and/or *Ureaplasma*) were detected by genus-specific, conventional semi-nested PCR targeting the 16S rRNA gene [52]. The primary PCR targeted a 700 bp portion of the 16S rRNA gene using primers GPO-1 and MGSO [52]. PCR was performed under the following conditions: 40 cycles of 94°C for 30 s, 64°C for 30 s, and 72°C for 60 s. The secondary PCR used primers My-ins [53] and MGSO, and 2 μl of the primary PCR product as template. Thermocycling parameters included 35 cycles of 94°C for 30 s, 60°C for 30 s, and 72°C for 60 s. *Ureaplasma* species (*U*. *parvum* and *U*. *urealyticum*) were detected using a conventional PCR based on the multiple-banded antigen gene with primers UMS-125 and UMA226, which yield products of two different sizes depending on the target species: 403 bp (*Ureaplasma parvum*) or 443 bp (*Ureaplasma urealyticum*) [54, 55]. Representative PCR products (several products of each size generated) were sequenced and confirmed to the expected target sequence. Every PCR assay contained a no template control (NTC) reaction containing all the PCR components without added template DNA and had to generate no signal (qPCR) or product (conventional PCR) for the assay to be valid.

### *cpn*60 universal target (UT) PCR and pyrosequencing

Generation of *cpn*60 PCR amplicon libraries for microbial profiling of vaginal samples was carried out as previously described [28]. *cpn*60 UT-specific primer sets [44, 56] were modified at the 5' end with a unique decamer multiplexing identification (MID) sequences. Amplicons were pooled in equimolar concentrations to create libraries for sequencing on the GS Junior platform as per the manufacturer’s recommendations (Roche/454, Brandford, CT, USA).

### Analysis of operational taxonomic units (OTU)

Sequence data were processed as previously described with minor modifications [28], using the default on-rig procedures from 454/Roche, which include confirmation of key sequence and trimming for quality. To classify experimental data, MID-partitioned reads were mapped using Bowtie 2 (http://bowtie-bio.sourceforge.net/bowtie2/) on to a previously created, manually curated reference set of 1,561 OTU sequences generated by assembly of *cpn*60 sequence reads from each of 546 vaginal microbiome samples from non-pregnant and pregnant Canadian women of reproductive age. The reference assembly was created by the microbial Profiling Using Metagenomic Assembly pipeline (mPUMA, http://mpuma.sourceforge.net)[57] with Trinity as the assembly tool [58]. Assembled OTU (> 150 bp) were identified and labeled according to their nearest reference sequence determined by watered-Blast comparison [21] to the *cpn*60 reference database, cpnDB_nr_vag (downloaded on September 18, 2014 from http://www.cpndb.ca, [59]). OTU having less than 55% identity to any reference sequence were considered to be non-*cpn*60 sequences and removed from the data set [60]. This reference assembly strategy facilitates comparison of microbiome profiles from various cohorts under study, including the 310 women described in this sub-study. Raw sequence data files for the 310 samples described in this study were deposited to the NCBI Sequence Read Archive (Accession SRP056439, BioProject PRJNA278895).

### Statistical analysis

All analyses were carried out in R v3.1.1 (R Core Team 2014). We restricted the pool of OTU to those having a total of at least 20 reads across all samples. A Jensen-Shannon distance matrix was calculated using the ‘vegdist’ function in the *vegan* package [61], with a custom distance function that calculates the square root of the Jensen-Shannon divergence [62]. This distance matrix was used for hierarchical clustering using the ‘hclust’ function in R, with Ward linkage.

Several internal cluster validation metrics were assessed [63], including average silhouette width [64], average Pearson gamma [65] and Dunn index [66], as calculated by the ‘cluster.stats’ function in the *fpc* package [67]. The Pearson gamma index measures the correlation between distances and a vector of 0s and 1s where 0 indicates the same cluster, 1 indicates a different cluster and higher values indicate better fit to the data [65]. The optimal cluster number was validated using a bootstrapping technique, as implemented by the ‘clusterboot’ function in the *fpc* package, with 500 bootstrap replicates for each of 5, 6, 7, 8, 9 and 10 clusters. Once the optimal number of clusters was determined, taxonomic composition was compared to previously published CST [18, 27]. Relationships between cluster membership and the variables listed in Table 1 were assessed using Fisher exact tests. All p-values were corrected for multiple testing using the Benjamini-Hochberg method with a false discovery rate of 0.05 [68]. Principal coordinates analysis from all the OTU in the dataset were calculated from Bray-Curtis dissimilarity values as the mean value of 100 subsampling of 1000 reads (or all reads available when less than 1000) in QIIME with the jackknifed_beta_diversity.py command [69].

**Table 1:** Sociodemographic, clinical and microbiological characteristics in relation to community state type (CST).

		Community State Type (CST)						
Characteristic	Descriptive statistic^a^	I (N = 156)	III (N = 50)	V (N = 22)	IVA (N = 36)	IVC(N = 22)	IVD (N = 24)	Sig.^b^
**Age (Mean ± SD, Range)**	30.1 ± 7.6 (18.6–49.2)							NS
18–25	111 (36%)	51 (33%)	22 (44%)	11 (50%)	13 (36%)	7 (32%)	7 (29%)	
26–35	131 (42%)	73 (47%)	17 (34%)	7 (32%)	12 (33%)	10 (46%)	12 (50%)	
36–49	68 (22%)	32 (21%)	11 (22%)	4 (18%)	11 (31%)	5 (23%)	5 (21%)	
**Body mass index (Mean ± SD, Range) (n = 307)**	23.9 ± 5.2 (15–50)							NS
Underweight (<18.5)	18 (5.8%)	4 (3%)	7 (14%)	1 (5%)	1 (3%)	2 (9%)	3 (13%)	
Normal weight (18.5–24.9)	194 (62.6%)	104 (67%)	27 (54%)	14 (64%)	27 (75%)	10 (45%)	12 (50%)	
Overweight (25.0–29.9)	55 (17.7%)	28 (18%)	10 (20%)	2 (9%)	5 (14%)	5 (23%)	5 (21%)	
Obese (>30)	40 (12.9%)	20 (13%)	5 (10%)	4 (18%)	3 (8%)	4 (18%)	4 (17%)	
**Ethnicity**								p = 0.05
White	200 (64.5%)	112 (72%)	24 (48%)	15 (68%)	19 (53%)	16 (73%)	14 (58%)	
Asian	60 (19.4%)	22 (14%)	17 (34%)	4 (18%)	9 (25%)	1 (5%)	7 (29%)	
South Asian	12 (3.9%)	4 (3%)	2 (4%)	2 (9%)	2 (6%)	2 (9%)	-	
Black	9 (2.9%)	5 (3%)	-	-	2 (6%)	1 (5%)	1 (4%)	
Aboriginal	6 (1.9%)	2 (1%)	1 (2%)	1 (5%)	1 (3%)	-	1 (4%)	
Other/Mixed Ethnicity	19 (6.1%)	11 (7%)	6 (12%)	-	3 (8%)	2 (9%)	1 (4%)	
**Education**								NS
Some high school/Graduated	97 (31.3%)	43 (28%)	18 (36%)	9 (41%)	10 (28%)	8 (41%)	9 (38%)	
Some post-secondary/Degree	153 (49.4%)	79 (51%)	25 (50%)	8 (36%)	18 (33%)	12 (54%)	11 (25%)	
Graduate Degree	60 (19.4%)	34 (22%)	7 (14%)	5 (23%)	8 (22%)	2 (9%)	4 (17%)	
**Current smoker**	39 (12.6%)	18 (12%)	8 (16%)	4 (18%)	2 (6%)	3 (14%)	4 (17%)	NS
**Menstrual cycle frequency (n = 309)**								NS
Regular (3–5 weeks)	228 (73.5%)	117 (75%)	37 (74%)	18 (82%)	24 (67%)	15 (68%)	17 (71%)	
Irregular (>5 weeks)	50 (16.1%)	22 (14%)	9 (18%)	3 (13%)	10 (28%)	3 (14%)	3 (13%)	
Altered by contraception	31 (10.0%)	16 (10%)	4 (8%)	1 (5%)	2 (6%)	4 (18%)	4 (17%)	
**Days since last menstrual period (median, range)**	17.5 (0–3653)							NS
7 days or fewer		15 (10%)	4 (8%)	1 (5%)	4 (11%)	2 (9%)	2 (8%)	
8–30 days		116 (74%)	39 (78%)	16 (73%)	22 (61%)	14 (64%)	18 (75%)	
31–60 days		16 (10%)	3 (6%)	4 (18%)	4 (11%)	1 (5%)	1 (4%)	
>60 days		9 (6%)	4 (8%)	1 (5%)	6 (17%)	5 (23%)	3 (13%)	
**Used tampons in past 2 months**	208 (67.1%)	114 (73%)	31 (62%)	16 (73%)	20 (56%)	15 (68%)	12 (50%)	NS
**Feminine hygiene products** ^**a**^								NS
Used in past 2 months	25 (8.1%)	10 (6%)	6 (12%)	1 (5%)	3 (8%)	2 (9%)	3 (13%)	
Used in past 48 hours	8 (2.6%)	2 (1%)	2 (4%)	1 (5%)	1 (3%)	1 (5%)	1 (4%)	
**Number of sexual partners in past year (Mean ± SD, range)**	1.5 ± 1.9 (0–25)							NS
None		19 (12%)	4 (8%)	1 (5%)	12 (33%)	3 (14%)	2 (8%)	
1		99 (63%)	37 (74%)	18 (82%)	20 (56%)	12 (54%)	12 (50%)	
2–3		27 (17%)	6 (12%)	1 (5%)	1 (3%)	4 (18%)	8 (33%)	
4 or more		11 (7%)	3 (6%)	2 (9%)	3 (8%)	3 (14%)	1 (4%)	
**Number of sexual partners in past 2 months (Mean ± SD, range)**	0.8 ± 0.6 (0–4)							NS
None		37 (23%)	10 (20%)	2 (9%)	15 (42%)	8 (41%)	6 (25%)	
1		114 (73%)	38 (76%)	19 (86%)	21 (58%)	12 (50%)	17 (71%)	
2 or more		5 (3%)	2 (4%)	1 (5%)	-	2 (9%)	-	
**Vaginal intercourse in past month**	222 (71.6%)	113 (72%)	39 (78%)	20 (91%)	21 (58%)	13 (59%)	16 (67%)	NS
Used condoms	122 (39.4%)	63 (40%)	22 (44%)	9 (46%)	15 (42%)	7 (32%)	6 (25%)	
**Vaginal intercourse in past 48 h**	44 (14.2%)	17 (11%)	10 (20%)	5 (23%)	5 (14%)	3 (14%)	4 (17%)	NS
Used condoms	21 (6.8%)	8 (5%)	5 (10%)	2 (9%)	4 (11%)	1 (5%)	1 (4%)	
**Contraceptive use**								NS
Combined Estrogen/Progestin^b^	89 (28.7%)	44 (28%)	16 (32%)	10 (45%)	6 (17%)	4 (18%)	9 (38%)	
Progestin-based^c^	37 (11.9%)	18 (12%)	4 (8%)	2 (9%)	4 (11%)	5 (23%)	4 (17%)	
Copper IUD	21 (6.8%)	9 (6%)	6 (12%)	2 (9%)	-	3 (14%)	1 (4%)	
**Lifetime history of sexually transmitted infection(s)**	52 (16.8%)	29 (19%)	8 (16%)	3 (13%)	3 (8%)	2 (9%)	7 (29%)	NS
Chlamydia	22 (7.1%)	11 (7%)	2 (4%)	2 (9%)	2 (6%)	1 (5%)	4 (17%)	
Gonorrhoea	4 (1.3%)	1 (1%)	-	-	1 (3%)	1 (5%)	1 (4%)	
Genital Herpes (HSV)	12 (3.9%)	7 (4%)	3 (6%)	-	1 (3%)	-	1 (4%)	
Genital Warts (HPV)	21 (6.8)	12 (8%)	3 (6%)	2 (9%)	1 (3%)	1 (5%)	2 (8%)	
**Self-reported vaginal symptoms** ^**d**^ **(n = 305)**								NS
In past two weeks	41 (13.2%)	20 (13%)	3 (6%)	5 (23%)	6 (17%)	3 (14%)	4 (17%)	
In past 48 hours	24 (7.7%)	9 (6%)	3 (6%)	3 (13%)	4 (11%)	2 (9%)	3 (13%)	
**Nugent category (n = 307)**								p<0.0001
Not consistent with BV (0–3)	250 (80.6%)	151 (97%)	45 (90%)	21 (42%)	22 (61%)	7 (32%)	4 (17%)	
Intermediate BV (4–6)	25 (8.1%)	4 (3%)	3 (6%)	1 (5%)	10 (28%)	3 (14%)	4 (17%)	
Consistent with BV (7–10)	32 (10.3%)	-	-	-	4 (11%)	12 (50%)	16 (67%)	
**Estimated bacterial load (total copies of 16S rRNA gene)**								NS
10^4^ or less		24 (15%)	7 (14%)	3 (13%)	8 (22%)	2 (9%)	4 (17%)	
10^5^−10^6^		50 (32%)	19 (38%)	5 (23%)	9 (25%)	5 (23%)	9 (38%)	
10^7^−10^8^		78 (50%)	23 (46%)	14 (64%)	16 (44%)	11 (50%)	4 (17%)	
10^9^ or more		4 (3%)	1 (2%)	-	3 (8%)	4 (18%)	7 (29%)	
**Presence of Mollicutes**	217 (70%)	106 (68%)	35 (70%)	13 (59%)	27 (75%)	19 (86%)	17 (71%)	NS
**Presence of *Ureaplasma***	149 (48%)							NS
*U. parvum*		64 (41%)	21 (42%)	8 (36%)	13 (36%)	10 (45%)	11 (25%)	
*U. urealyticum*		7 (4%)	4 (8%)	-	3 (8%)	3 (14%)	4 (17%)	
Both		-	-	-	1 (3%)	-	-	
**Shannon diversity (Mean ± SD)**	1.68 ± 1.14	1.32 ± 1.00	1.65 ± 0.95	1.76 ± 0.91	2.49 ± 1.62	2.15 ± 0.74	2.31 ± 0.91	p<0.0001
**Chao1 richness (Mean ± SD)**	48 ± 26	45 ± 29	50 ± 21	44 ± 19	58 ± 33	45 ± 15	49 ± 16	NS

^a^ Continuous variables are reported as means ± 95% CI (range). Categoric variables are reported as N (%).

^b^ p value is shown for significant relationships. NS = not significant.

Variation in OTU abundance according to Nugent score and clinical/demographic variables was assessed using center log ratio transformations in ALDEx2 (v2.0.7.2), an algorithm for compositional analysis that uses a Dirichlet-multinomial model to infer relative abundances from counts [70]. Expected p-values for Kruskal-Wallis tests of differences among clinical data categories were determined using 128 Monte Carlo runs. A false discovery rate of 0.05 was applied using the Benjamini-Hochberg correction and adjusted p-values are reported [71]. Shannon diversity and Chao1 estimated species richness were calculated as the mean of 100 subsampling of 1000 reads (or all reads available when less than 1000) in QIIME with the multiple_rarefactions.py, alpha_diversity.py and collate_alpha.py commands [69]. These were compared to CST and clinical/demographic variables using linear models for Shannon diversity and Poisson regressions for Chao1 richness estimates. Significance was assessed using likelihood-ratio tests with p-values corrected for multiple testing using the Benjamini-Hochberg method as above.

### Phylogenetic analysis

For abundance tree analysis, 164 full-length *cpn*60 UT sequences that are nearest neighbours for all OTU with abundance exceeding 1% of total reads in at least a single individual were aligned using ClustalW with gap opening/gap extension penalties of 10/0.2 and phylogenetic trees constructed by neighbour-joining with 100 bootstrap iterations in MEGA v6 (www.megasoftware.net) [72]. Proportions of total reads for subgroups of women were represented as circles whose combined area is equal to 100% of all reads for that subgroup, as previously described [22]. Each subgroup of women is represented as a different colour based on Nugent score or CST, with all individual libraries normalized to the same total read count.

## Results

### Cohort description

Five hundred and thirty-four women were initially recruited, referred, or contacted the study coordinating centre to participate in the study. Potential participants were provided with detailed information about the study and screened to ensure they met eligibility criteria, resulting in 320 women who attended study visits and consented to participate. Ten women were excluded post-enrollment due to erroneous assessment of exclusion criteria, inability to provide vaginal samples, or laboratory errors (e.g. lost/missing samples, improper storage of samples), resulting in total sample size of 310 women.

The average age of participants was 30 (Table 1). The majority of women had been sexually active and reported a history of predominantly heterosexual relationships, but some had bisexual and same sex partnerships. Of those who had been sexually active, most had been sexually active during the past year, with the number of partners ranging from 1–25. The ethnicity distribution of our study population was diverse and representative of Canadian populations from the White, Asian, Black, Aboriginal and Hispanic communities according to 2006 census data [73]. A subgroup of women (n = 24 or 7.7%) who self-identified as not having specific vaginal health issues reported vaginal symptoms (odour, abnormal discharge, and/or irritation) within 48 hours prior to of sample collection (Table 1). The majority of women had Nugent scores not consistent with BV (BV-, i.e. *Lactobacillus* morphotype-dominant); however, many women (57/310 or 18.4%) had Nugent scores categorized as BV-intermediate (BVI) or consistent with BV (BV+).

### Vaginal microbiome profiling

A total of 3,114,714 *cpn*60 reads were included in the analysis, and were mapped on to 1,437 unique OTU in the reference assembly (S1 Table). The average sequence read count was 10,047 per sample, with a median of 6,167 (range 195–120,487). Only 30 OTU were detected in at least 50% of women sampled (Table 2). The most prevalent and abundant OTU was OTU 1403: *L*. *crispatus* (99.8% identity), which was detected in 305/310 (98.4%) women and made up 36.75% of all reads. Interestingly, of the six additional OTU prevalent in over 90% of women, only OTU 1174: *L*. *iners* (100% identity) and OTU 1479: *L*. *jensenii* (95.7% identity) made up a significant proportion of the total reads (16.33% and 6.93%, respectively). The other four prevalent OTU, OTU 0490: *Atopobium vaginae* (94.1% identity), OTU 0026: *Streptococcus devriesei* (83.0% identity), OTU 1186: *L*. *acidophilus* (100% identity) and OTU 0021: *Weissella viridescens* (58.8% identity), all represented less than 0.3% of the overall reads mapped (0.20%, 0.29%, 0.32% and 0.17%, respectively) (Table 2). OTU 0026 and OTU 0021 had low percent identities to their nearest reference database matches (*Streptococcus devriesei* and *Weissella viridescens*, respectively) (Table 2), suggesting that these OTU represent as yet uncharacterized Firmicutes.

**Table 2:** Prevalence and proportion of total reads for OTU detected in at least half of study group.

Nearest neighbour^a^	OTU	% identity	Prevalence	% total reads
*Lactobacillus crispatus*	OTU 1403	99.8	305 (98%)	36.8
	OTU 1409	97.9	259 (84%)	1.6
	OTU 1197	97.5	229 (74%)	0.7
	OTU 1102	99.6	227 (73%)	0.7
*Lactobacillus jensenii*	OTU 1479	95.7	296 (96%)	7.0
	OTU 1182	100	162 (52%)	0.3
*Atopobium vaginae*	OTU 0490	94.1	293 (95%)	0.2
*Streptococcus devriesei*	OTU 0026	83.0	292 (94%)	0.3
*Lactobacillus acidophilus*	OTU 1186	100	286 (92%)	0.3
*Lactobacillus iners*	OTU 1174	100	285 (92%)	16.4
*Weissella viridescens*	OTU 0021	58.8	279 (90%)	0.2
*Desulfotalea psychrophila*	OTU 1048	76.1	251 (81%)	0.1
*Peptoniphilus harei*	OTU 0478	90.0	240 (77%)	0.07
*Clostridium innocuum*	OTU 0874	75.4	234 (76%)	0.08
*Streptococcus parasanguinis*	OTU 1396	95.9	234 (76%)	0.08
*Gardnerella vaginalis* subgroup A	OTU 1670	99.6	231 (75%)	7.1
*Gardnerella vaginalis* subgroup C	OTU 1668	99.3	231 (75%)	3.9
	OTU 1673	98.8	192 (62%)	0.1
*Prevotella tannerae*	OTU 0157	79.6	230 (74%)	0.1
*Faecalibacterium prausnitzii*	OTU 1134	84.1	224 (72%)	0.2
*Lactobacillus gasseri*	OTU 1355	99.7	223 (72%)	0.2
	OTU 0161	97.7	161 (52%)	2.0
*Sphingobium yanoikuyae*	OTU 1275	99.3	218 (70%)	0.1
*Gardnerella vaginalis* subgroup B	OTU 1651	98.8	202 (65%)	0.3
	OTU 1663	99.6	157 (51%)	2.6
*Massilia timonae*	OTU 0612	82.0	179 (58%)	0.03
*Acidaminococcus fermentans*	OTU 0182	78.7	173 (56%)	0.03
*Megasphaera* sp. genomsp. type 1	OTU 0193	83.2	161 (52%)	1.8
*Prevotella timonensis*	OTU 1380	97.7	157 (51%)	1.7

^a^Closest match in the cpnDB reference database based on sequence identity.

Total bacterial abundance per sample was estimated using qPCR of total 16S rRNA gene targets, while presence of Mollicutes in general and *Ureaplasma* species specifically were assessed by conventional PCR (Table 1). Estimates of total bacterial load ranged over more than five orders, from <10^4^ to >10^9^ 16S rRNA copies per sample. Mollicutes were detected in vaginal samples of 217/310 (70%) women, with 149/310 (48%) testing positive for *Ureaplasma* spp. *U*. *parvum* was detected more frequently (127/310 = 41%) than *U*. *urealyticum* (21/310 = 7%).

### Hierarchical clustering and principal components analysis of community state types (CST)

Hierarchical clustering of vaginal microbiome profiles resulted in six clusters with good support (S1 Fig). All Jaccard indices from bootstrapping for six clusters were greater than 0.6, with two clusters greater than 0.65, one cluster greater than 0.8 and two clusters greater than 0.9. When the number of clusters was reduced or increased, the Jaccard indices fell below 0.6, and in some cases, below 0.5. Average silhouette width and the Pearson gamma index were also highest for six clusters, and the Dunn index at five and six clusters, indicating the most overall support for six clusters (S1 Fig).

**Fig 1:**
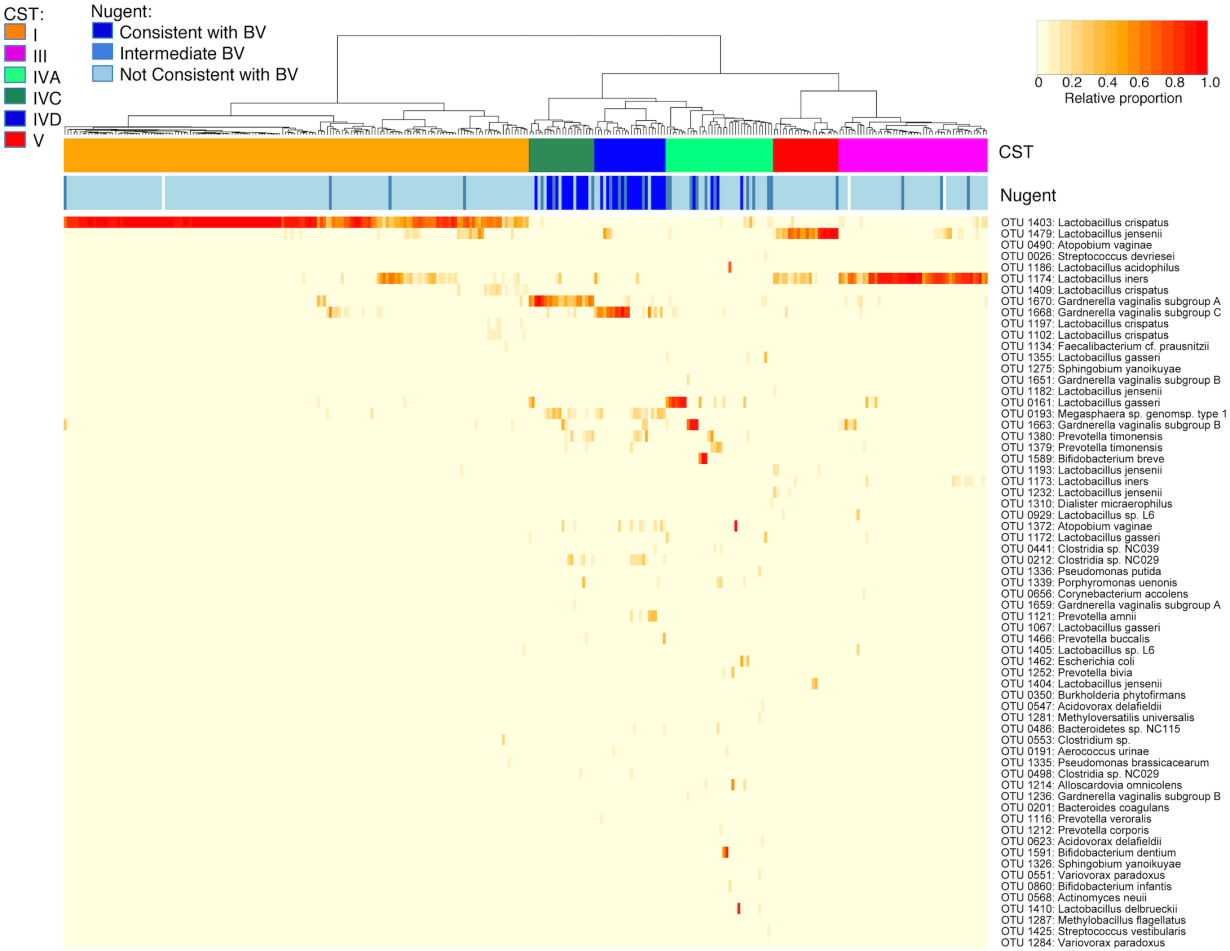
Vaginal microbiome profiles of Canadian women. Heatmap representing hierarchical clustering of Jensen-Shannon distance matrices with Ward linkage on the relative proportions of reads for each OTU within individual vaginal samples collected from healthy, reproductive-aged Canadian women (n = 310). Each column represents a woman’s vaginal microbiome profile, and each row represents an OTU. For clarity, only the top 65 OTU by read abundance are shown. The proportion of the total microbiome comprised is indicated in the yellow to red colour scheme. Community state type (CST) and Nugent category (Nugent) for each woman are indicated by the top bars.

*Lactobacillus*-dominated CST identified correspond to those previously described in 16S rRNA deep sequencing studies (Fig 1). Consistent with previous studies of healthy reproductive-aged women, the profiles of most women (228/310 = 74%) belonged to CST dominated by one of three *Lactobacillus* species, including CST I (OTU 1403: *L*. *crispatus* (99.8% identity), n = 156), CST III (OTU 1174: *L*. *iners* (100% identity), n = 50) and CST V (OTU 1479: *L*. *jensenii* (95.7% identity), n = 22). Most individuals in CST I had very high proportions of *L*. *crispatus* OTU and very few OTU from other species, with a minority of individuals with co-dominant *L*. *iners* or *L*. *jensenii* (S2 Fig, panel A). Although profiles from most women in CST III were almost exclusively composed of *L*. *iners*, several also had co-dominant populations of *L*. *jensenii*, *Gardnerella* subgroup B or *Gardnerella* subgroup A (S2 Fig, panel B). In contrast, *L*. *jensenii* was highly dominant in only 7/22 women in CST V, with *L*. *iners* co-dominant in most profiles (S2 Fig, panel C). As expected, most women in CST I, III and V had BV- Nugent scores (217/228 or 95%), and none had BV+ Nugent scores.

The *L*. *gasseri-*dominated CST II described by Ravel et al. [18] did not form its own supported cluster in this study, but *L*. *gasseri* was dominant in 7/36 or 19% of women in CST IVA (S2 Fig, panel D). Two other women in this cluster had profiles dominated by *Lactobacillus*, including one with *L*. *delbrueckii* and another with *L*. *acidophilus*. As described previously, CST IVA is remarkably heterogeneous relative to other CST, with profiles dominated by organisms besides *Lactobacillus*, including *Gardnerella* subgroup B (n = 4), *Bifidobacterium breve* (n = 3), *B*. *dentium* (n = 2) or *Atopobium vaginae* (n = 1), or with no single dominant OTU but even mixtures of *Stapylococcus*, *Streptococcus*, *Prevotella*, *Alloscardovia*, *Gardnerella* and *Lactobacillus* (n = 17). Although most of the women in this CST had BV- Nugent scores, 25% (8/36) had BVI Nugent scores.

For the remaining CST, IVC (S2 Fig, panel E) and IVD (panel F), both have dominant *Gardnerella* and sub-populations of Clostridiales and Bacteroidales. However, two distinct clusters based on OTU from different *cpn*60-defined *G*. *vaginalis* subgroups [47] were observed: one characterized by *Gardnerella* subgroup A (OTU 1670, 99.6% identity, n = 22) and the other by *Gardnerella* subgroup C (OTU 1668, 99.3% identity, n = 24). For women in CST IVC, *Gardnerella* subgroup A was dominant in most profiles, with sub-dominant populations of *Megasphaera* sp. and *Prevotella timonensis* in several women. Only about half of the women in CST IVD were dominated by *Gardnerella* subgroup C, while the other half had lower levels of this organism and co-dominant mixtures of *Megasphaera* sp., *Prevotella timonensis*, *P*. *amnii* and *Atopobium vaginae*. As expected, most of the women in CST IVC and IVD had BV+ Nugent scores (10/22 or 45% for IVC, 16/24 or 67% for IVD).

### Abundance of reads for each CST in phylogenetic context

**Fig 2:**
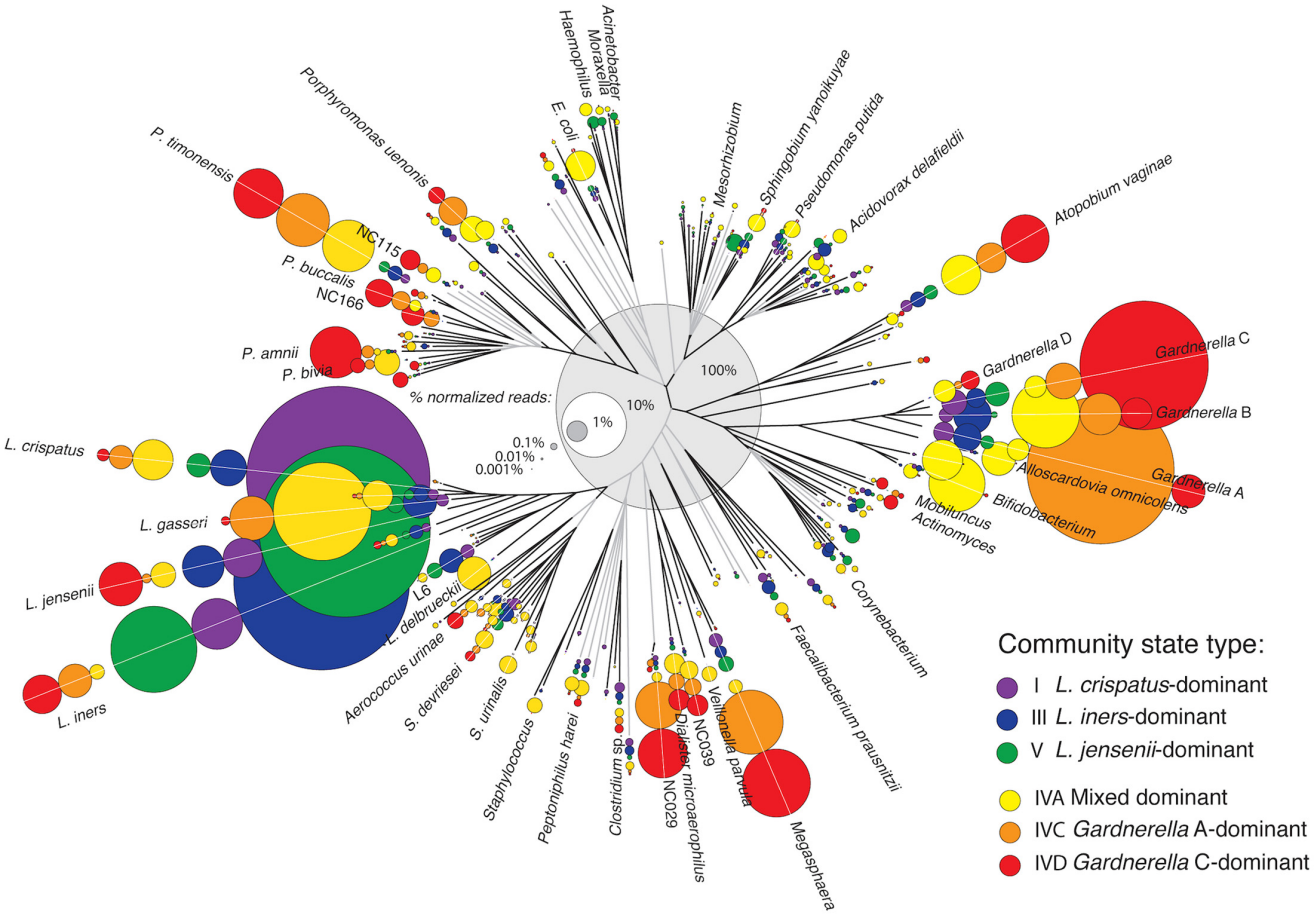
Abundance and phylogenetic relationships of *cpn*60 defined species detected in samples from each CST. Neighbour-joining phylogeny of 164 unique *cpn*60 universal target using MEGA v6 for Mac, that are nearest neighbours for OTU detected at an abundance of at least 1% of at least one woman's sample. Branches proceeding from nodes with less than 50% bootstrap percent (100 replicates) are shown in grey. Circle area represents the proportion of each taxon (branch) in total normalized reads from women in different CST. Therefore, the combined area of all circles of the same colour equals the area of the central grey circle that represents 100% of reads attributed to that CST. Only the most abundant taxa are labelled. *L*. = *Lactobacillus*, *P*. = *Prevotella*, *E*. = *Escherichia*

In order to better visualize sub-dominant populations in relation to CST, microbiome sequence data was viewed in phylogenetic context (Fig 2). CST I, III and V are clearly dominated by *L*. *crispatus*, *L*. *iners* and *L*. *jensenii* respectively. CST IVC and IVD are clearly dominated by *Gardnerella* subgroup A and C respectively. However these organisms represent a smaller proportion of total reads for these CST compared to the *Lactobacillus*-dominated CST. The most commonly observed organisms are found in all CST, confirming that fluctuations in relative abundance rather than presence or absence of specific organisms characterize CST.

Several sub-dominant populations are virtually absent in *Lactobacillus*-dominated CST (I, III, V), including *Gardnerella* subgroup D and *Prevotella bivia*, while *Lactobacillus* sp. L6 is virtually absent in *Gardnerella*-dominant CST (IVC, IVD). *Prevotella timonensis* and *Atopobium vaginae* are present at similar high levels in CST IVA, IVC and IVD and similar low levels in CST I, III and V, while *Clostridium* sp. NC029 and *Megasphaera* sp. are similarly abundant in CST IVC and IVD and lower in CST I, III, V and IVA. A few sub-dominant taxa are evenly distributed across all CST.

Many organisms were observed in relatively high proportions in CST IVA, reflecting the mixed dominant phenotype. *L*. *gasseri*, *Gardnerella* subgroup B, *Bifidobacterium* sp., *Atopobium vaginae*, and *E*. *coli* sequences were all most abundant in IVA, which was expected since all individuals with profiles dominated by these organisms are included in this CST. Interestingly, abundance of several sub-dominant organisms from the phyla Firmicutes, Actinobacteria and Proteobacteria was associated with CST IVA, to a lesser extent with other, *Lactobacillus*-dominated CST, and virtually absent in CST IVC and IVD. These observations indicate that broad phylogenetic groups of organisms are more likely to be present at low-abundance in women with *Lactobacillus*-dominated CST or CST IVA compared to IVC or IVD.

### CST in relation to socio-behavioural variables

Despite extensive investigation of factors previously reported to influence the vaginal microbiota profile, ethnicity was the only variable significantly related to CST when considering White and Asian women only, perhaps due to low numbers of women representing other ethnic groups (Table 1, S3 Fig). CST membership was associated with Asian vs. White ethnicity (Benjamini-Hochberg adjusted p = 0.049) with greater than expected numbers of CST III in Asian women (S3 Fig). There were no other significant relationships between CST membership and clinical or demographic variables (Table 1).

### CST and specific OTU in relation to Nugent score

**Fig 3:**
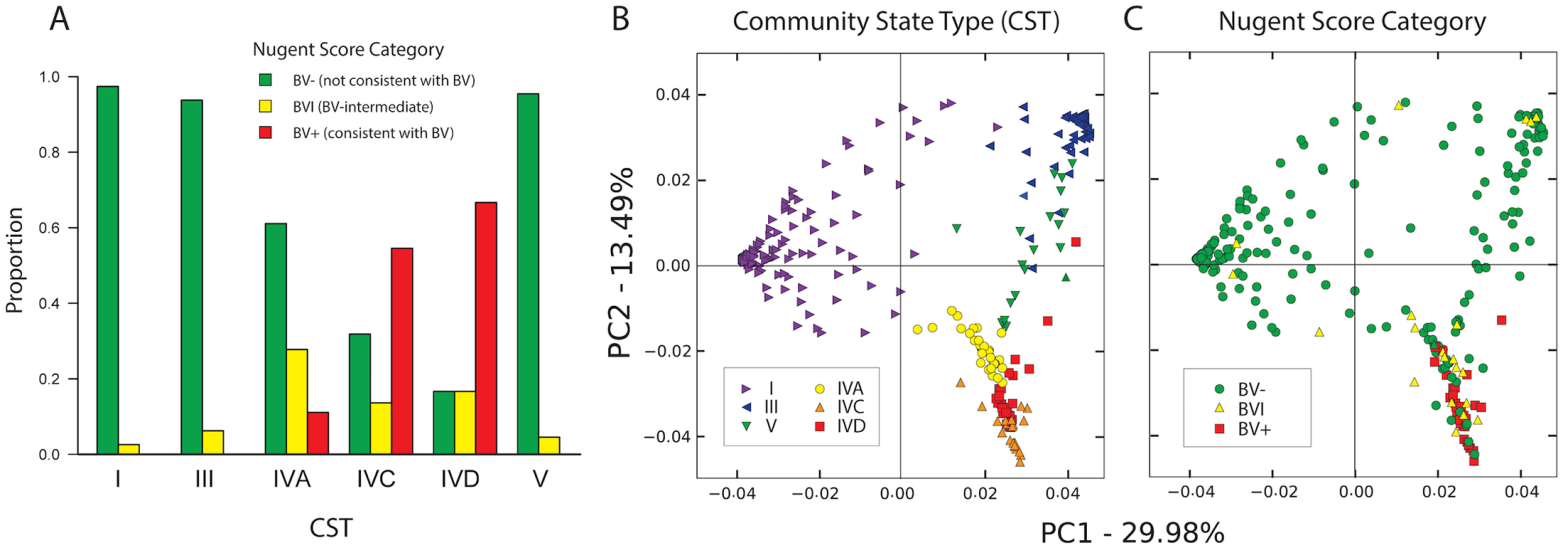
Correspondence between CST and Nugent score. (A) Significant (Fisher exact test, Benjamini-Hochberg adjusted p < 0.0001) relationship between CST and Nugent score, including predominance of CST I, III and V in BV- samples, and CST IVC and IVD in BV+ samples. BVI samples were observed most frequently in CST IVA. (B) Principal components of microbiome for all members of study group, with individuals coloured by CST, and C) Nugent score.

The relationship between CST and Nugent score, noted in the analysis of heatmaps above, was determined to be statistically significant (Fisher exact test, Benjamini-Hochberg adjusted p < 0.0001; Table 1), with greater than expected numbers of profiles in CST IVC and IVD having BV+ Nugent scores, and greater than expected numbers of profiles in CST IVA having BVI scores (Fig 3A). Principal component analysis of all samples according to CST (Fig 3B) and Nugent score (Fig 3C) revealed extensive overlap between the two categories, with BV- Nugent scores and BVI Nugent scores observed in all CST, while BV+ Nugent scores were observed only in CST IVA, IVC and IVD. Of the 24 women who self-identified as having vaginal symptoms within 48 hours prior to sample collection (Table 1), only three had Nugent scores consistent with BV and each belonged to a different CST group (IVA, IVC and IVD, respectively) (S4 Fig).

**Fig 4:**
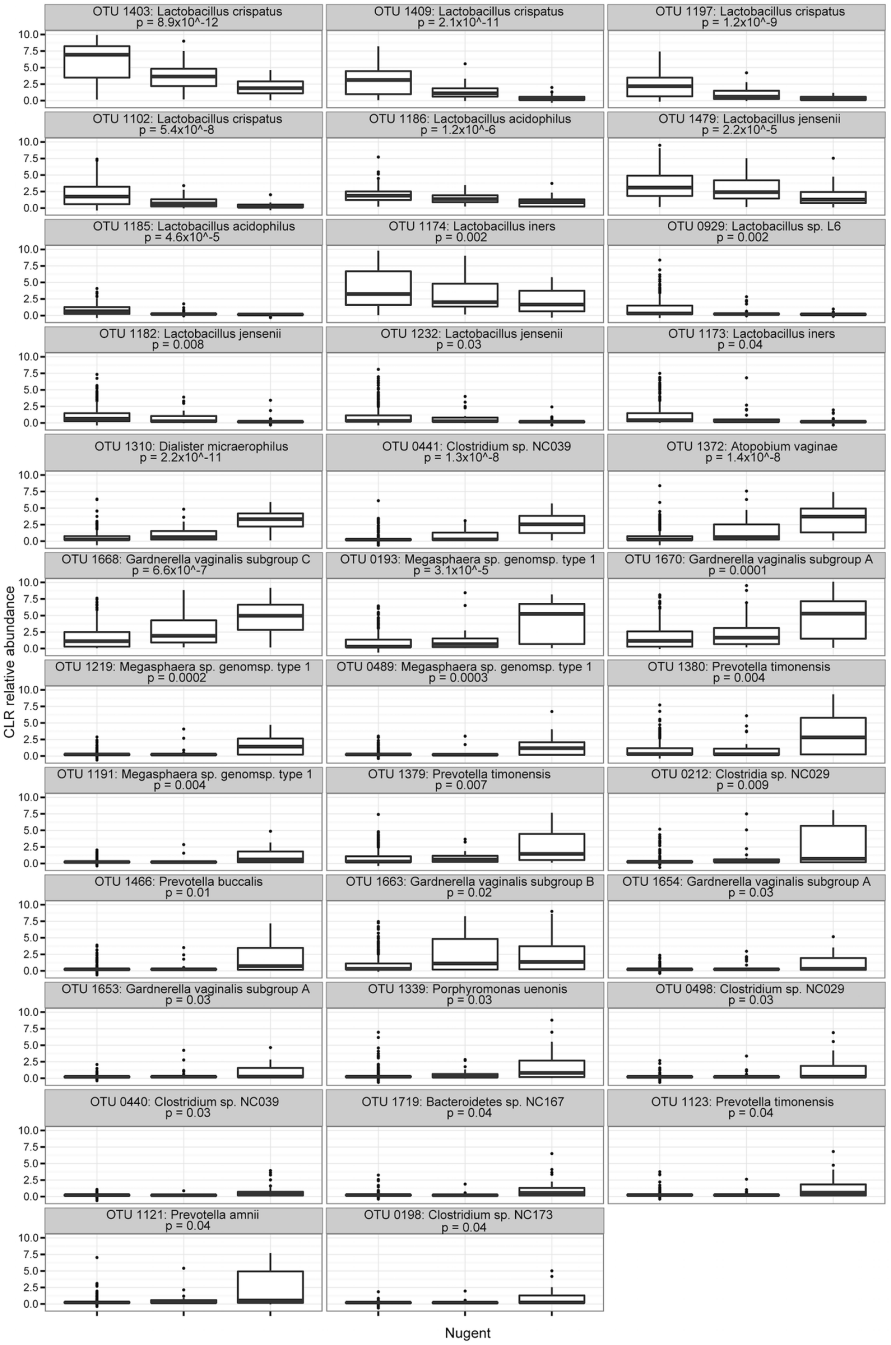
OTU associated with BV-, BVI and BV+ categories. Boxplots showing differences in relative abundance (as center log transformed counts) among Nugent categories across 35 OTU that were significantly different among Nugent categories from the ALDeX analysis. These plots show all reads +1 so that zero values could be included in the log transformation. All p-values shown are corrected by the Benjamini-Hochberg method as indicated in the text.

A total of 35 OTU were significantly different across Nugent categories by Kruskal-Wallis test. Not surprisingly, all OTU with a lower abundance in BVI or BV+ Nugent categories were *Lactobacillus* spp., while the remainder were all present at a higher abundance in those with BVI and/or BV+ Nugent scores (Fig 4). OTU 1663: *G*. *vaginalis* subgroup B was somewhat higher in the BVI category, although this was not statistically significant. No significant relationship between Mollicutes detection (by targeted PCR) and Nugent score was observed. A phylogenetic tree illustrating relative abundance of organisms in relation to Nugent score category was constructed (S5 Fig), reinforcing previous observations regarding CST IVA in the CST abundance tree (Fig 2). Several sub-dominant organisms in the phyla Actinobacteria and Proteobacteria were observed almost exclusively in BV- and BVI samples, and several *Streptococcus* sp. were observed almost exclusively in BVI samples.

## Discussion

The vaginal microbiota of 310 non-pregnant women aged 18 to 49 years was consistent with previous studies from Canada and other countries that sampled as few as 32 and as many as 494 adolescent girls and/or women [15–19, 27]. The ethnic diversity in our study cohort reflects the Canadian population, with relatively large proportions of White and Asian women and smaller proportions of Black, Aboriginal and Hispanic subjects. Microbiota profiles in this cohort were largely characterized by the dominance of a single OTU most closely related to *Lactobacillus* species including *L*. *crispatus*, *L*. *iners*, *L*. *jensenii*, and *L*. *gasseri*. However, we also observed CST dominated by two of the four known *Gardnerella* subgroups [47, 48]. Individuals with dominant *Gardnerella* subgroups A and C separated into CST IVC and IVD, respectively, while those with dominant *Gardnerella* subgroup B clustered within the heterogeneous CST IVA category. Overall, this *cpn*60-based study confirms CST defined in earlier studies using the 16S rRNA target, while expanding the range of CST that can be defined due to increased resolution of *cpn*60. The ability to target *Gardnerella* subgroups in the context of microbial community profiles may help improve our clinical understanding of the role played by these organisms in the vaginal microbiome.

Similar to findings in our study, the majority of vaginal bacterial CST in other studies have been found to be dominated by lactobacilli, although the particular pattern of dominance of any one species varies among reports. This is perhaps not surprising given the variety of cohorts studied and the techniques used (S2 Table). In our study, CST groups I, III and V were dominated by *L*. *crispatus*, *L*. *iners* and *L*. *jensenii* respectively, but *L*. *gasseri* did not define any of the CST, although it was dominant in 7/36 (19%) of women in CST IVA. This species has been reported to define CST in previous T-RFLP [15] or 16S rRNA amplicon sequencing studies [17, 18, 27], but has also been found using the same methods as dominant or co-dominant in a subset of profiles within larger CST [16], or as a low abundance organism [19]. In this study, CST IVA was similar to other described groups, including a wide range of anaerobic species. Equivalent CST from other studies include “group IV” (*Atopobium vaginae*, *Gardnerella vaginalis* and *Prevotella)* [16], “group IV” (*Prevotella*, *Dialister*, *Atopobium* and others) [18], “group IVB” (*Atopobium*, *Prevotella* and others) [27], and “group IV” (*Atopobium* and others) [17]. Further groups have been defined containing *Streptococcus* spp. (“group VII”) and high numbers of clones from a single clade of the family Lachnospiraceae (“group VIII”) [15]. *Atopobium* is common to all of these previous descriptions, and was equally represented in CST IVA, IVB and IVC in this study (Fig 2).

A noteworthy difference between our results and those of other reports is the identification of two distinct CST dominated by *Gardnerella*: IVC and IVD, dominated by *Gardnerella* subgroups A and C respectively. Drell et al. [19] identified a *Gardnerella* dominated CST (CST IV) in their recent study of Estonian women, but in other studies *Gardnerella* occurs as a sub-dominant constituent of one or more CST, e.g. CST IV in [18] or CST IVB in [27]. The variation in reports of abundance and prevalence of *Gardnerella* in the vaginal microbiome of healthy reproductive aged women can be explained by differences in the detection of these sequences with various “universal” PCR primers for a variety of gene targets. However, the evidence from real-time quantitative PCR validation of *cpn*60 pyrosequencing read abundance for *Gardnerella* performed previously by our group suggests that the higher estimates provided by *cpn*60-based microbiome profiles are realistic [28]. Regardless of abundance, the discrimination of two distinct community types containing either *Gardnerella cpn*60-defined subgroups A or C, and the association of *Gardnerella* subgroup B with the BVI communities in our study is intriguing since these subgroups have been shown to be phenotypically and genomically distinct [47, 48] and differentially associated with BV status [49]. Further studies of the biological and clinical significance of this diverse taxon are currently underway.

Analysis of CST composition in a phylogenetic context (Fig 2) offers several additional insights, including the ability to visualize group-level differences as well as low- and medium-abundance sequences that correspond to the lightest colours in heatmaps. Several low-abundance genera were observed most frequently in CST IVA (e.g. *Streptococcus* spp.) and/or in *Lactobacillus*-dominated CST (several Proteobacteria and Actinobacteria). These findings confirm previous observations in a *cpn*60-based study of women in Nairobi, Kenya, where Proteobacteria genera nearest *Acidovorax* and *Sphingobium* were associated with *Lactobacillus*-dominated profiles [22]. These organisms may be less likely to be detected using 16S rRNA gene sequencing [21], which would account for these components of the vaginal microbiota not being widely reported previously.

Reports of the prevalence of Mollicutes in reproductive aged women vary widely, and these reports are invariably based on culture and/or screening of samples with targeted PCR assays such as those used in the current study. Mollicutes are frequently undetected in 16S rRNA based studies due to bias in “universal” PCR primers [74] and they have not been detected previously in the vaginal microbiota using the *cpn*60 UT, due to the absence of the target sequence in almost all Mollicutes species [20–22, 28]. Our observation that 70% of women in our study cohort were PCR positive for Mollicutes is consistent with the high end of reported values [75].

The range of total 16S rRNA copies per sample was remarkable, including samples with fewer than 10^4^ to more than 10^9^ copies. However, no significant relationship between bacterial population size and CST was detected (Table 1). While variations in 16S rRNA copy number per genome among different taxa and variations in the amount of sample material collected with the swabs may have contributed to this finding, it also reflects the enormous variation in microbial population density that is possible. Bacterial overgrowth, as evidenced on clue cells, is certainly associated with clinical BV, but our results suggest that even among healthy, asymptomatic women, there is large variation in density of the vaginal microbiota. Similar observations have been made using flow cytometry [76].

Our ability to associate OTU sequences assembled from *cpn*60 UT amplicon libraries with those of known bacterial species depends upon the availability of representative reference sequences. In the particular case of BVAB1 and BVAB2, this is problematic since these organisms have not been cultured to date and are known only by their 16S rRNA sequences [77]. We detected several clostridia-like OTU, such as OTU 0441: *Clostridium* sp. NC039 (79.8% identity), OTU 0440: *Clostridium* sp. NC039 (80% identity), OTU 0402: *Clostridium* sp. NC039 (80.4% identity) or OTU 0553: *Clostridium* sp. (64.6% identity), OTU 0207: *Clostridium* sp. (81.6% identity) that could possibly correspond to BVAB1 and BVAB2. However, identification of the *cpn*60 sequences of BVAB1 and BVAB2 awaits either culture or whole genome sequencing of these fastidious organisms.

Our results confirm a previously reported difference between the vaginal microbiota of Asian and White women in North America [18]. No obvious explanation can be given for this difference. No other socio-demographic, hygiene or behavioural practices showed any significant correlation with CST. This is perhaps affected by minimal variations in behavioural and hygiene practices in a healthy Canadian cohort, as well as the cross-sectional design of the study. Future comparisons with different cohorts of women are needed to address this question further.

At the CST level, the only significant factors besides ethnicity were Nugent score category and Shannon’s diversity (highest in CST IVA), while at the OTU level, Nugent score was the only significant factor. Although the associations between *Lactobacillus*-dominated CST I, III and V with BV- Nugent scores and *Gardnerella*-dominated CST IVC and IVD with BV+ Nugent scores are straightforward, the link between the mixed dominant CST IVA and BVI Nugent scores is more difficult to draw. In the PCA plots, BVI samples and CST IVA clearly overlap with each other (Fig 3), however BVI samples are also observed in all other CST. In the abundance tree analysis, *Streptococcus*, *Staphylococcus*, *Corynebacterium* and *Gardnerella* subgroup B among others were more abundant in both CST IVA and BVI samples (Fig 2 and S6 Fig). Since CST IVA is significantly associated with BVI Nugent scores, these observations may provide insight into the ambiguous clinical significance of the intermediate BV Nugent score category.

In conclusion, this large study of Canadian women has provided a solid foundation for expanded investigation of the vaginal microbiota into clinically significant cohorts. Comparisons of this cohort with HIV-positive non-pregnant women, non-pregnant women with recurrent vulvovaginitis, pregnant women at high and low risks of complications are currently in progress. The overall agreement of CST composition in healthy Canadian women to women from other parts of the world reaffirms that vaginal microbiota research is converging on a broad understanding of microbial community membership in this body site. This makes it easier for research groups to share their investigative findings and collectively seek to improve the diagnosis of aberrant conditions and more appropriately treat them with existing and novel therapeutics. The challenge for future work will be to tease apart the details of this community structure, in concert with social and behavioural information, to understand and effect positive clinical outcomes for women’s health.

## Supporting Information

S1 Fig Validation of CST. Cluster validation scores generated by average silhouette width, Pearson gamma and Dunn index results. The highest value for each method (six clusters) indicates the strongest support for that number of clusters in the data.
(PDF)

S2 Fig Detailed CST profiles. Hierarchical clustering of Jensen-Shannon distances with Ward linkage on the relative proportions of reads for each OTU within women from each CST cluster. Each column represents a woman’s vaginal microbiome profile, and each row represents an OTU. For clarity, only the top 65 OTU by read abundance are shown on the heatmap. The proportion of total sequence for each OTU is indicated in the yellow to red colour scheme. Nugent category for each woman is indicated by the top bar (light blue = BV-, medium blue = BVI, dark blue = BV+). A: Cluster I, B: Cluster III, C: Cluster IVA, D: Cluster IVC, E: Cluster IVD, F: Cluster V
(PDF)

S3 Fig Ethnicity and CST. Distribution of CST among Asian and White women in this study. CST membership was associated with Asian vs. White ethnicity (Benjamini-Hochberg adjusted p = 0.049) with greater than expected numbers of CST III in Asian women.
(PDF)

S4 Fig BV+ microbiome profiles. Hierarchical clustering of Jensen-Shannon distance matrices with Ward linkage on the relative proportions of reads for each OTU within women with Nugent scores consistent with BV (scores 7–10) (n = 32). Each column represents a woman’s vaginal microbiome profile, and each row represents an OTU. For clarity, only the top 65 OTU by read abundance are shown on the heatmap. The proportion of the total microbiome comprised of each OTU is indicated in the yellow to red colour scheme. Community state type (CST) and whether vaginal symptoms (odor, abnormal discharge, and/or irritation) were self-reported within 48 hours of sample collection (Symptoms 48 hr) for each woman is indicated by the top bars.
(PDF)

S5 Fig Nugent score abundance tree. Abundance and phylogenetic relationships of *cpn*60 defined species detected in samples from each Nugent score category. Neighbour-joining phylogeny of 164 unique *cpn*60 universal target using MEGA v6 for Mac, that are nearest neighbours for OTU detected at an abundance of at least 1% of at least one woman's sample. Branches proceeding from nodes with less than 50% bootstrap percent (100 replicates) are shown in grey. Circle area represents the proportion of each taxon (branch) in total normalized reads from women in different Nugent categories. Most abundant taxa are labeled. L. = *Lactobacillus*, P. = *Prevotella*, E. = *Escherichia*
(PDF)

S1 Table Summary of OTU analyzed in this study. Best database match, taxonomic lineage of the best database match and abundance in each library are shown.
(XLSX)

S2 Table Previous culture-independent studies reporting Community State Types. (DOCX)
