# Supplementary figures and images for: A Study of the Vaginal Microbiome in Healthy Canadian Women Utilizing cpn60-Based Molecular Profiling Reveals Distinct Gardnerella Subgroup Community State Types
Source: PLoS One. 2015 Aug 12;10(8):e0135620. doi: 10.1371/journal.pone.0135620 (PMC4534464; doi:10.1371/journal.pone.0135620)

**average silhouette width**

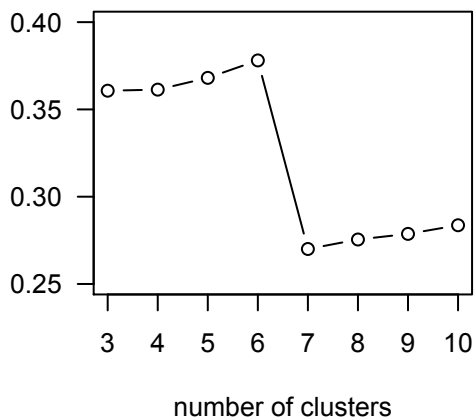

**dunn index**

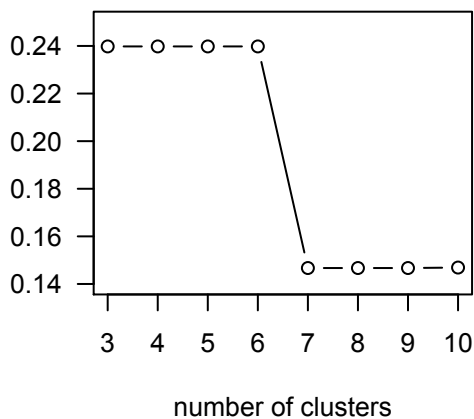

**pearson gamma**

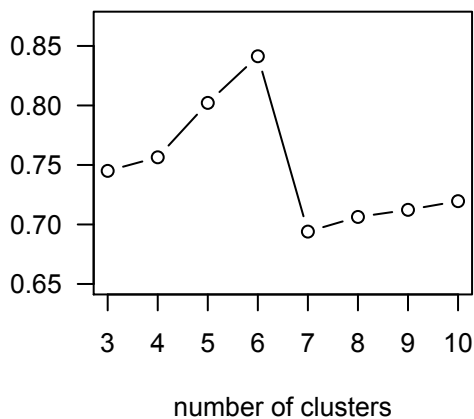

Supplement: S1 Fig — Cluster validation scores generated by average silhouette width, Pearson gamma and Dunn index results. The highest value for each method (six clusters) indicates the strongest support for that number of clusters in the data. (PDF) [file pone.0135620.s001.pdf]

A. CST I

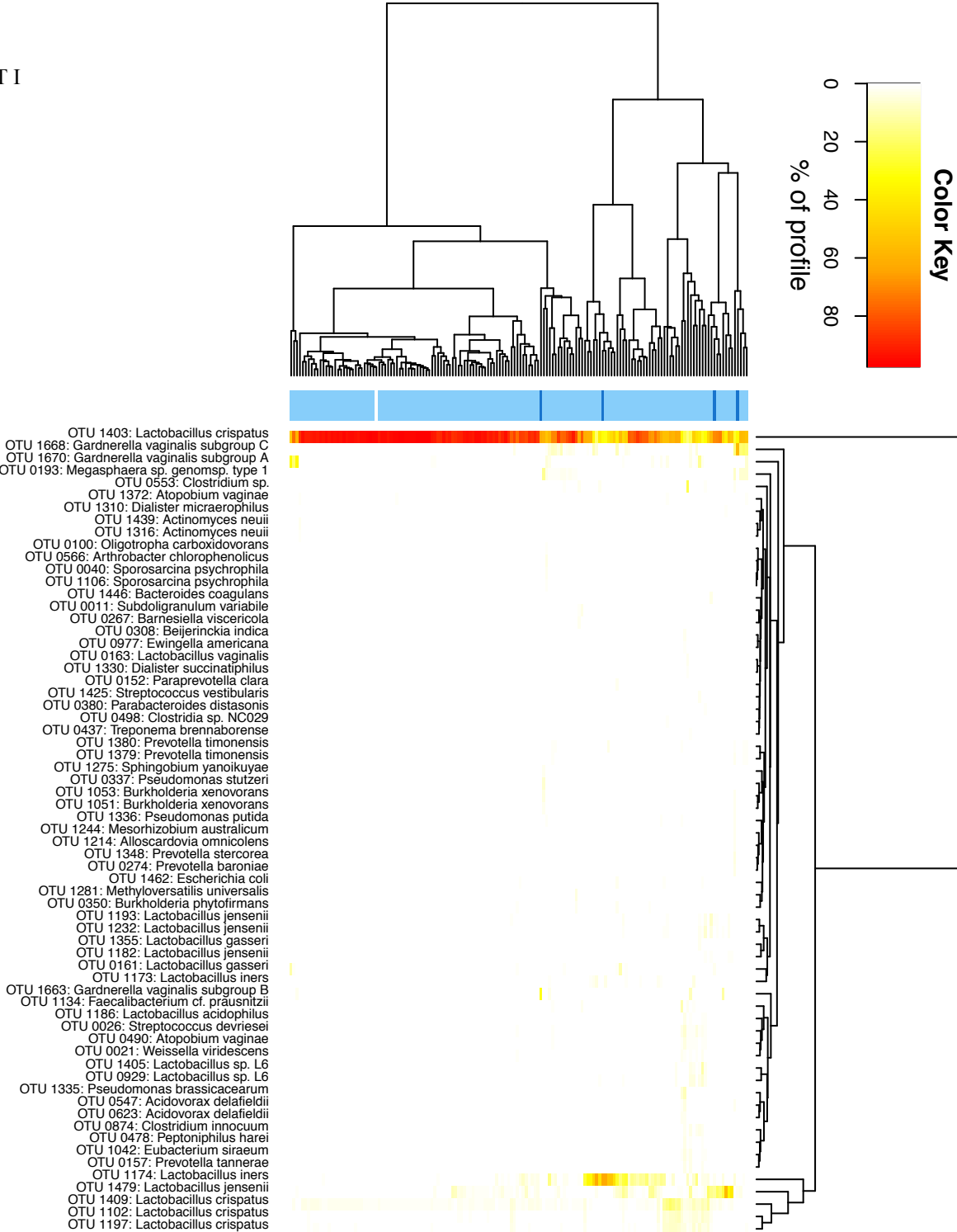

B. CST II

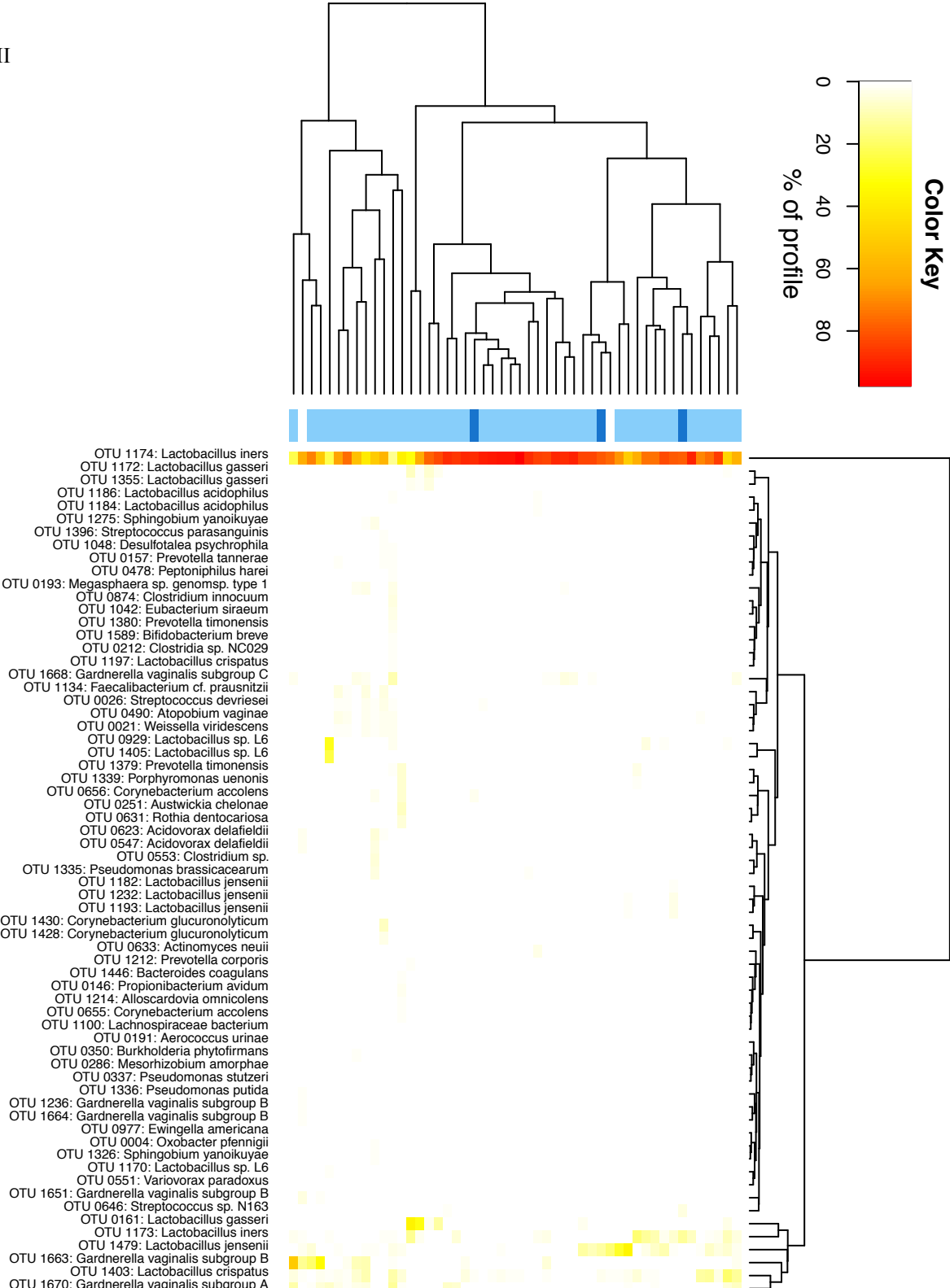

# C. CST III

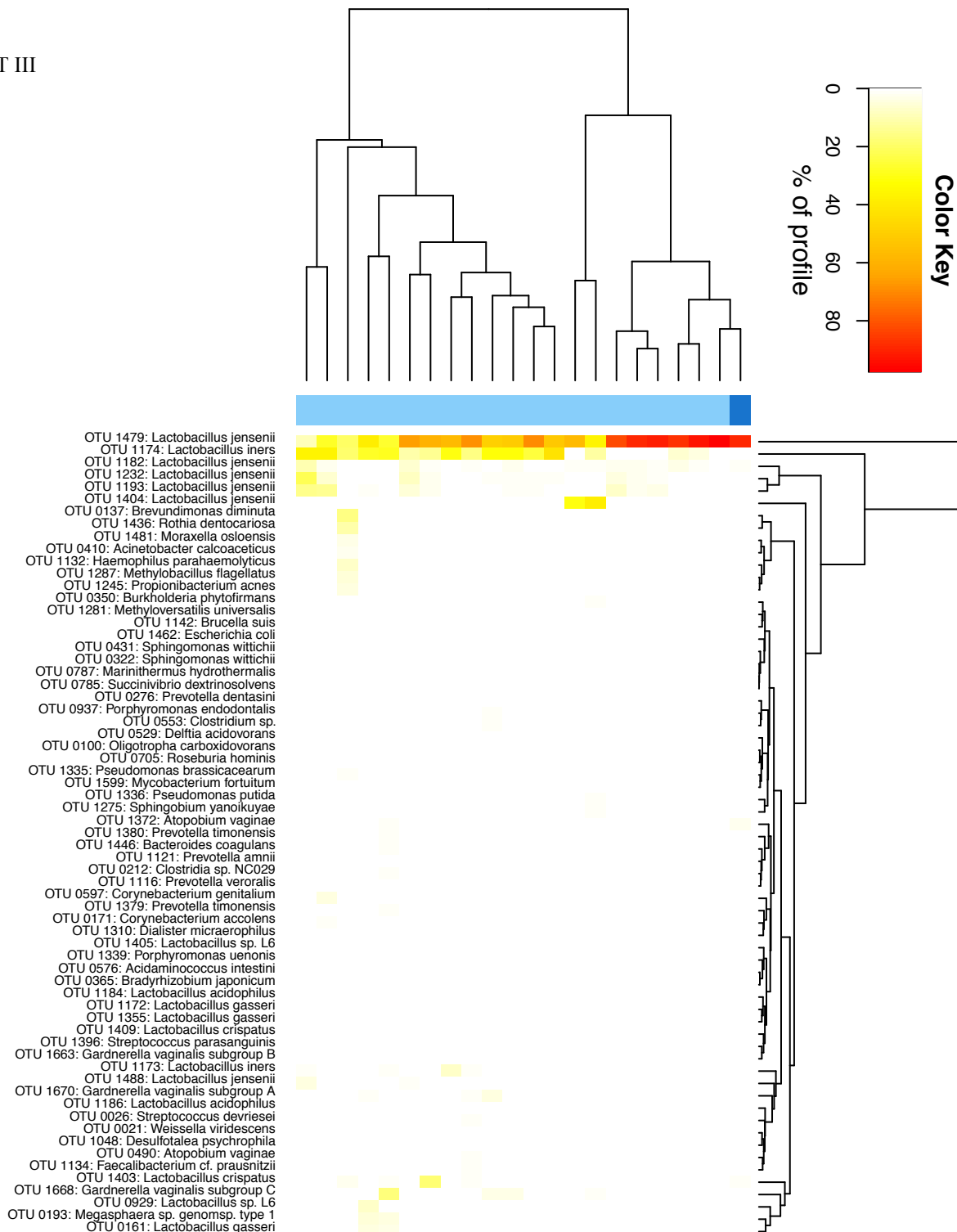

D. CST IVA

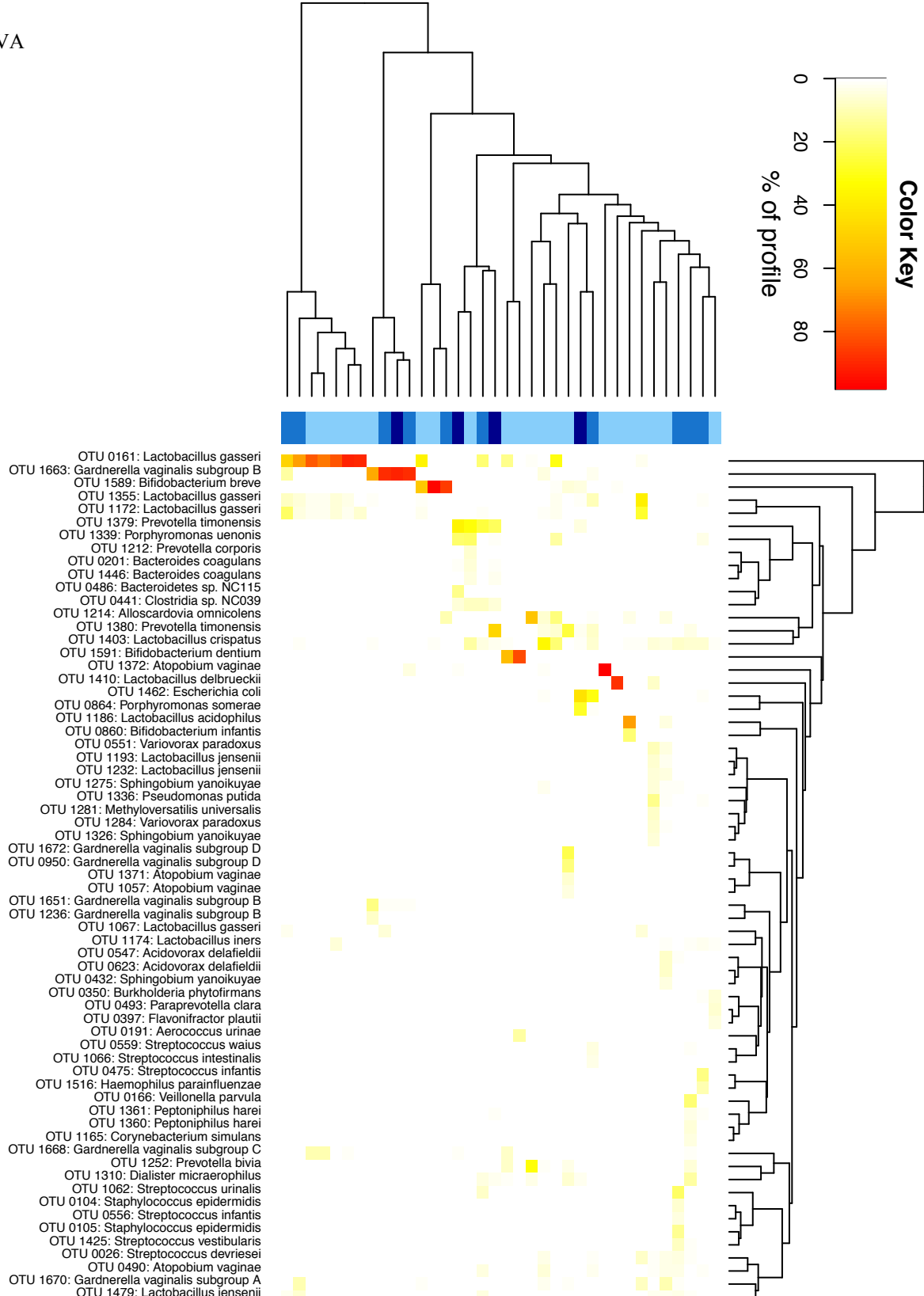

E. CST IVB

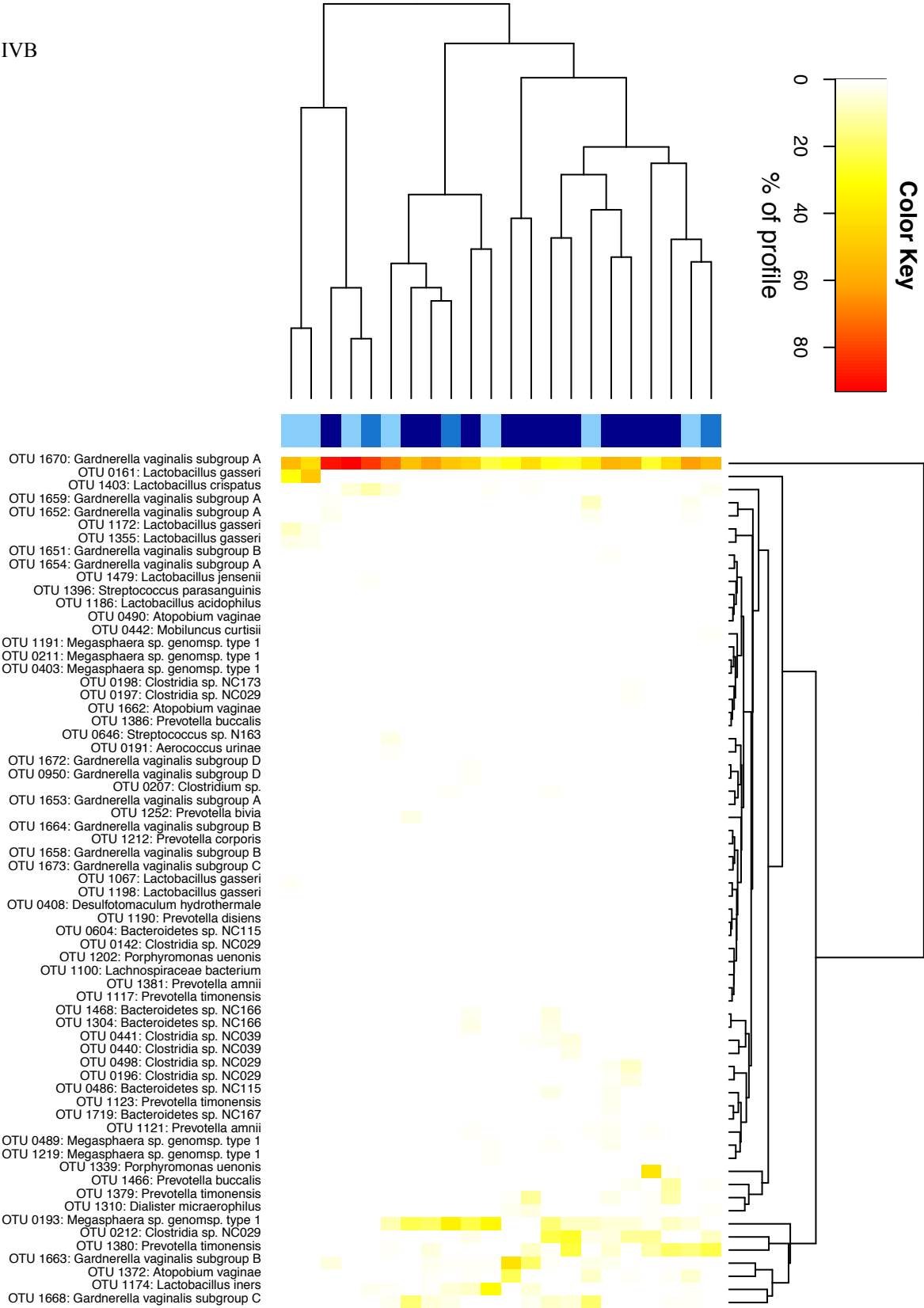

F. CST IVC

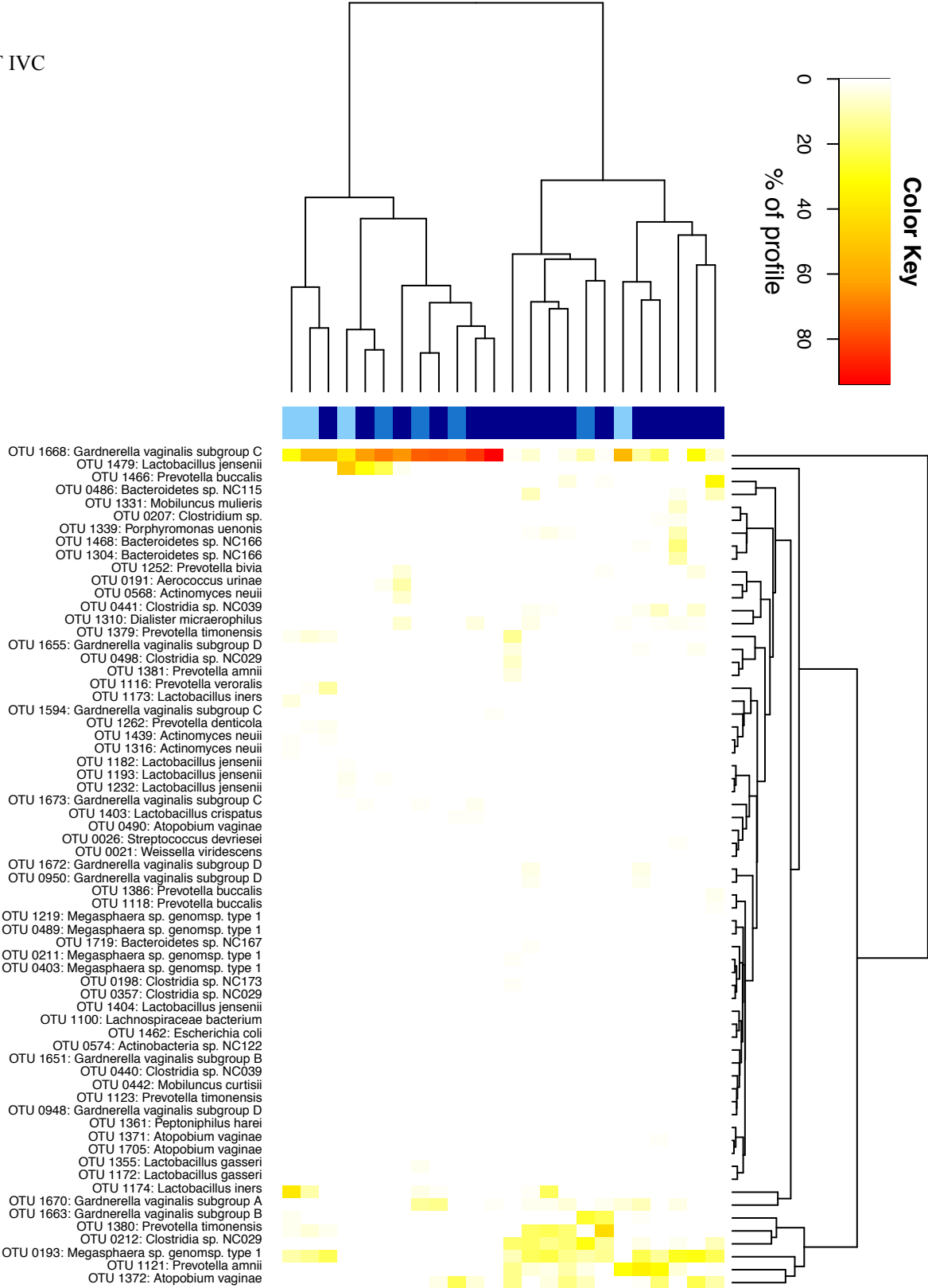

Supplement: S2 Fig — Hierarchical clustering of Jensen-Shannon distances with Ward linkage on the relative proportions of reads for each OTU within women from each CST cluster. Each column represents a woman’s vaginal microbiome profile, and each row represents an OTU. For clarity, only the top 65 OTU by read abundance are shown on the heatmap. The proportion of total sequence for each OTU is indicated in the yellow to red colour scheme. Nugent category for each woman is indicated by the top bar (light blue = BV-, medium blue = BVI, dark blue = BV+). A: Cluster I, B: Cluster III, C: Cluster IVA, D: Cluster IVC, E: Cluster IVD, F: Cluster V (PDF) [file pone.0135620.s002.pdf]

**CST by Asian vs. White Ethnicity**

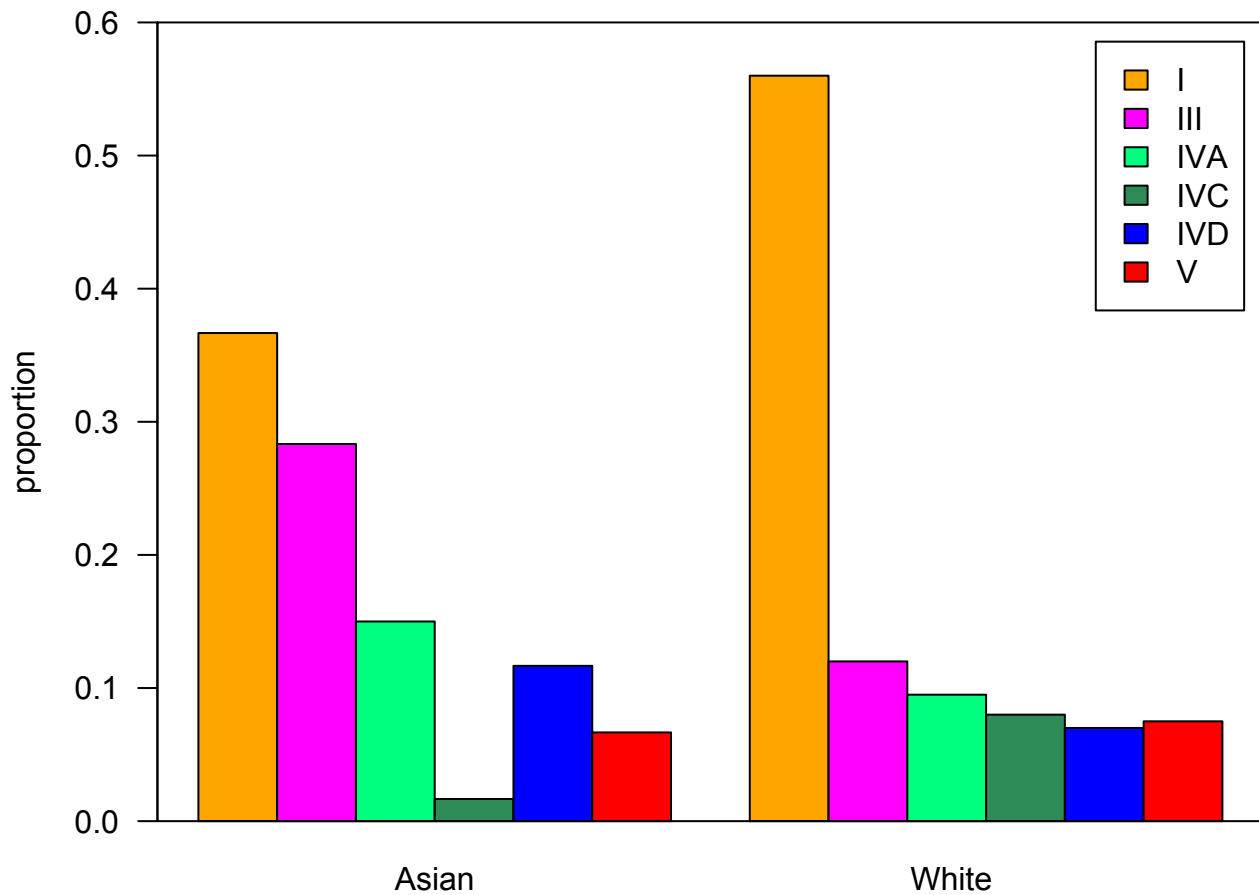

Supplement: S3 Fig — Distribution of CST among Asian and White women in this study. CST membership was associated with Asian vs. White ethnicity (Benjamini-Hochberg adjusted p = 0.049) with greater than expected numbers of CST III in Asian women. (PDF) [file pone.0135620.s003.pdf]

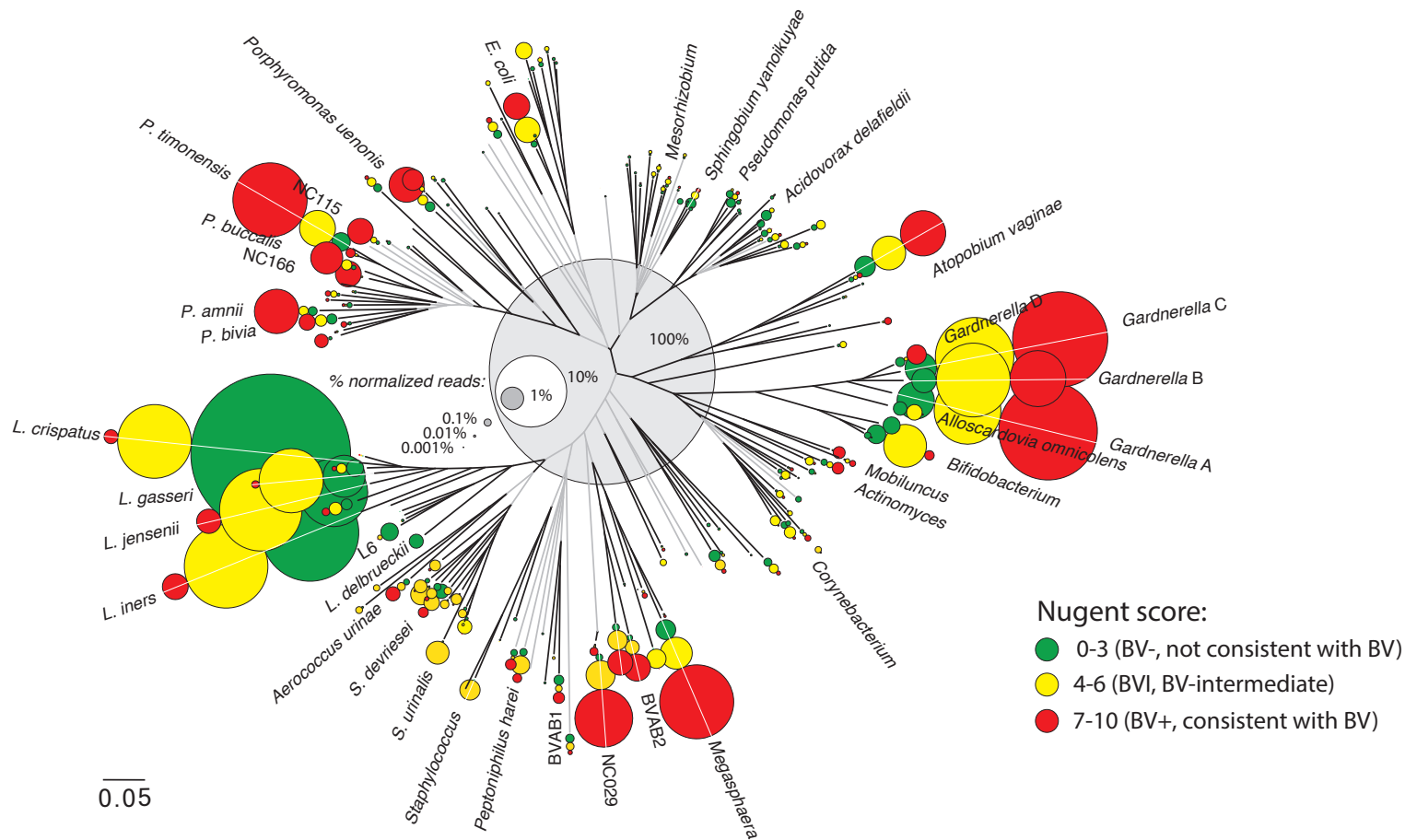

Supplement: S5 Fig — Abundance and phylogenetic relationships of cpn60 defined species detected in samples from each Nugent score category. Neighbour-joining phylogeny of 164 unique cpn60 universal target using MEGA v6 for Mac, that are nearest neighbours for OTU detected at an abundance of at least 1% of at least one woman's sample. Branches proceeding from nodes with less than 50% bootstrap percent (100 replicates) are shown in grey. Circle area represents the proportion of each taxon (branch) in total normalized reads from women in different Nugent categories. Most abundant taxa are labeled. L. = Lactobacillus, P. = Prevotella, E. = Escherichia (PDF) [file pone.0135620.s005.pdf]
