# Supplementary material for: A Study of the Vaginal Microbiome in Healthy Canadian Women Utilizing cpn60-Based Molecular Profiling Reveals Distinct Gardnerella Subgroup Community State Types
Source: PLoS One. 2015 Aug 12;10(8):e0135620. doi: 10.1371/journal.pone.0135620 (PMC4534464; doi:10.1371/journal.pone.0135620)
Supplement: S4 Fig — Hierarchical clustering of Jensen-Shannon distance matrices with Ward linkage on the relative proportions of reads for each OTU within women with Nugent scores consistent with BV (scores 7–10) (n = 32). Each column represents a woman’s vaginal microbiome profile, and each row represents an OTU. For clarity, only the top 65 OTU by read abundance are shown on the heatmap. The proportion of the total microbiome comprised of each OTU is indicated in the yellow to red colour scheme. Community state type (CST) and whether vaginal symptoms (odor, abnormal discharge, and/or irritation) were self-reported within 48 hours of sample collection (Symptoms 48 hr) for each woman is indicated by the top bars. (PDF) [file pone.0135620.s004.pdf]

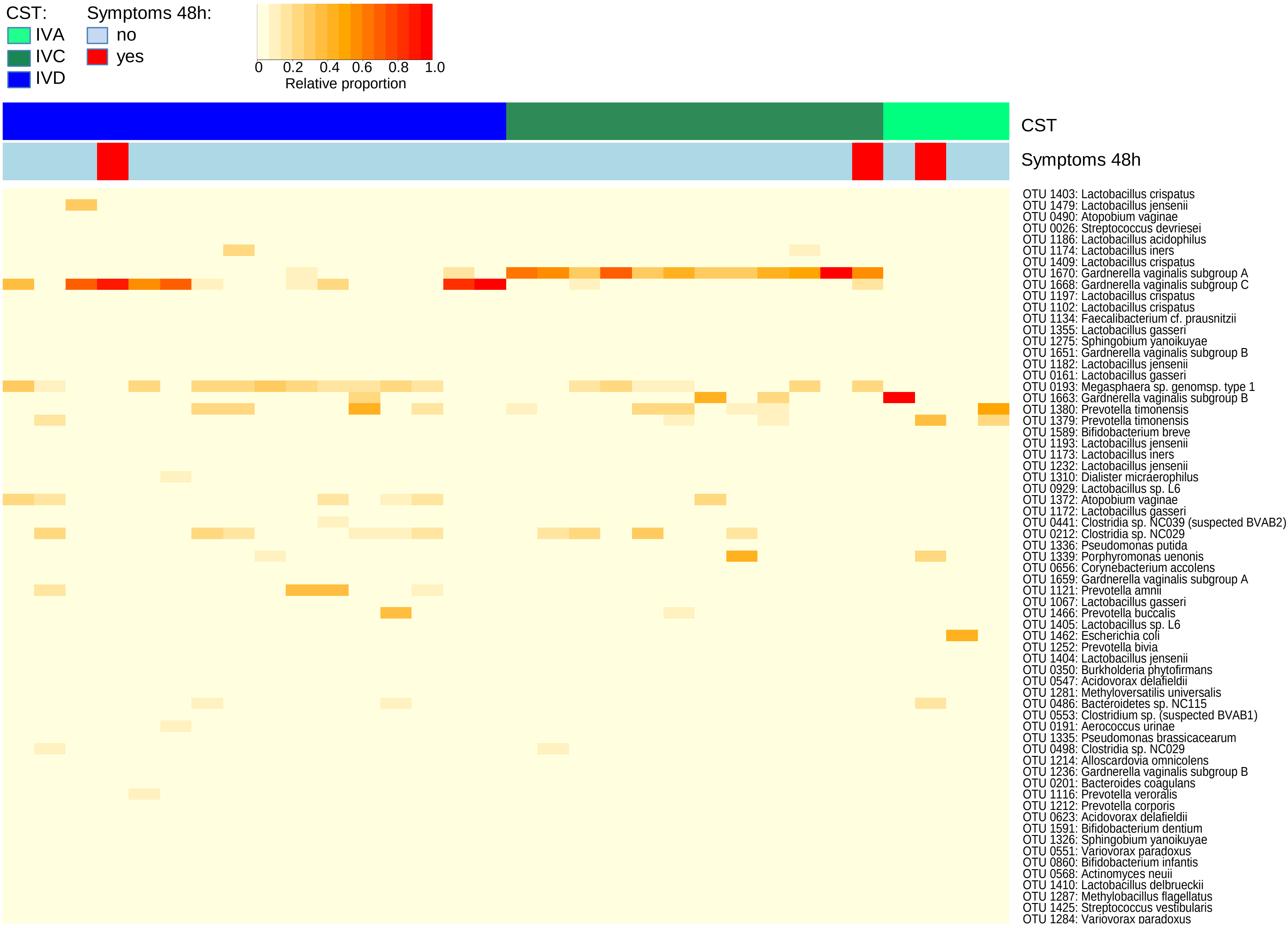
CST:
IVA
IVC
IVD
Symptoms 48h:
no
yes
0
0.2
0.4
0.6
0.8
1.0
Relative proportion
CST
Symptoms 48h
OTU 1403: Lactobacillus crispatus
OTU 1479: Lactobacillus jensenii
OTU 0490: Atopobium vaginae
OTU 0026: Streptococcus devriesei
OTU 1186: Lactobacillus acidophilus
OTU 1174: Lactobacillus iners
OTU 1409: Lactobacillus crispatus
OTU 1670: Gardnerella vaginalis subgroup A
OTU 1668: Gardnerella vaginalis subgroup C
OTU 1197: Lactobacillus crispatus
OTU 1102: Lactobacillus crispatus
OTU 1134: Faecalibacterium cf. prausnitzii
OTU 1355: Lactobacillus gasseri
OTU 1275: Sphingobium yanoikuyae
OTU 1651: Gardnerella vaginalis subgroup B
OTU 1182: Lactobacillus jensenii
OTU 0161: Lactobacillus gasseri
OTU 0193: Megasphaera sp. genomsp. type 1
OTU 1663: Gardnerella vaginalis subgroup B
OTU 1380: Prevotella timonensis
OTU 1379: Prevotella timonensis
OTU 1589: Bifidobacterium breve
OTU 1193: Lactobacillus jensenii
OTU 1173: Lactobacillus iners
OTU 1232: Lactobacillus jensenii
OTU 1310: Dialister micraerophilus
OTU 0929: Lactobacillus sp. L6
OTU 1372: Atopobium vaginae
OTU 1172: Lactobacillus gasseri
OTU 0441: Clostridia sp. NC039 (suspected BVAB2)
OTU 0212: Clostridia sp. NC029
OTU 1336: Pseudomonas putida
OTU 1339: Porphyromonas uenonis
OTU 0656: Corynebacterium accolens
OTU 1659: Gardnerella vaginalis subgroup A
OTU 1121: Prevotella amnii
OTU 1067: Lactobacillus gasseri
OTU 1466: Prevotella buccalis
OTU 1405: Lactobacillus sp. L6
OTU 1462: Escherichia coli
OTU 1252: Prevotella bivia
OTU 1404: Lactobacillus jensenii
OTU 0350: Burkholderia phytofirmans
OTU 0547: Acidovorax delafieldii
OTU 1281: Methyloversatilis universalis
OTU 0486: Bacteroidetes sp. NC115
OTU 0553: Clostridium sp. (suspected BVAB1)
OTU 0191: Aerococcus urinae
OTU 1335: Pseudomonas brassicacearum
OTU 0498: Clostridia sp. NC029
OTU 1214: Alloscardovia omnicolens
OTU 1236: Gardnerella vaginalis subgroup B
OTU 0201: Bacteroides coagulans
OTU 1116: Prevotella veroralis
OTU 1212: Prevotella corporis
OTU 0623: Acidovorax delafieldii
OTU 1591: Bifidobacterium dentium
OTU 1326: Sphingobium yanoikuyae
OTU 0551: Variovorax paradoxus
OTU 0860: Bifidobacterium infantis
OTU 0568: Actinomyces neuii
OTU 1410: Lactobacillus delbrueckii
OTU 1287: Methylobacillus flagellatus
OTU 1425: Streptococcus vestibularis
OTU 1284: Variovorax paradoxus
