# Supplementary material for: A Study of the Vaginal Microbiome in Healthy Canadian Women Utilizing cpn60-Based Molecular Profiling Reveals Distinct Gardnerella Subgroup Community State Types
Source: PLoS One. 2015 Aug 12;10(8):e0135620. doi: 10.1371/journal.pone.0135620 (PMC4534464; doi:10.1371/journal.pone.0135620)
Supplement: S2 Table — (DOCX) [file pone.0135620.s007.docx]

| **Study** | **Method** | **Country** | **No. of subjects** | **Age** | **Ethnicity** | **No. of CST and dominant species** |
| --- | --- | --- | --- | --- | --- | --- |
| Zhou *et al*. 2007 | T-RFLP | USA | 144 | 13-40 | 69 Black, 75 Caucasian | 8 CST: iners, crispatus, iners/crispatus, jensenii/crispatus, gasseri, 3 mixed |
| Yamamoto *et al*. 2009 | T-RFLP | USA | 90 | 13-18 | 36 Black, 54 Caucasian | 4 CST: iners, crispatus, gasseri/crispatus, mixed |
| Zhou *et al.* 2010 | 16S rRNA amplicon sequencing | Japan | 73 | 18-45 | Japanese | 7 CST: iners, crispatus, iners/crispatus, gasseri, jensenii, atopobium, mixed |
| Ravel *et al*. 2011 | 16S rRNA amplicon sequencing | USA | 396 | 12-45 | 104 Black, 98 Caucasian, 97 Asian, 97 Hispanic | 5 CST: iners, crispatus, gasseri, jensenii, mixed |
| Gajer *et al*. 2012 | 16S rRNA amplicon sequencing | USA | 32 | 18-40+ | 16 Black, 13 Caucasian, 1 Hispanic, 2 other | 5 CST: iners, crispatus, gasseri, 2 mixed |
| Drell *et al*. 2013 | 16S rRNA amplicon sequencing | Estonia | 494 | 15-44 | Caucasian | 6 CST: iners, crispatus, iners/crispatus, crispatus/gardnerella, gardnerella, mixed |
| This study | *cpn*60 amplicon sequencing | Canada | 310 | 19-49 | 9 Black, 200 Caucasian, 60 Asian, 12 South Asian, 6 Aboriginal, 4 Hispanic, 19 other | 6 CST: iners, crispatus, jensenii, Gardnerella A, Gardnerella C, mixed |

**S2 Table.** Summary of characteristics of previous culture-independent studies reporting Community State Types (CST).
